# Hyperexcitable interneurons trigger cortical spreading depression in an *Scn1a* migraine model

**DOI:** 10.1172/JCI142202

**Published:** 2021-11-01

**Authors:** Eva Auffenberg, Ulrike B.S. Hedrich, Raffaella Barbieri, Daniela Miely, Bernhard Groschup, Thomas V. Wuttke, Niklas Vogel, Philipp Lührs, Ilaria Zanardi, Sara Bertelli, Nadine Spielmann, Valerie Gailus-Durner, Helmut Fuchs, Martin Hrabě de Angelis, Michael Pusch, Martin Dichgans, Holger Lerche, Paola Gavazzo, Nikolaus Plesnila, Tobias Freilinger

**Affiliations:** 1 Department of Neurology and Epileptology, Hertie Institute for Clinical Brain Research, University of Tübingen, Tübingen, Germany.; 2 Institute for Stroke and Dementia Research (ISD), University Hospital, LMU Munich, Munich, Germany.; 3 Biophysics Institute, Consiglio Nazionale delle Ricerche (CNR), Genoa, Italy.; 4 Department of Neurosurgery, University of Tübingen, Tübingen, Germany.; 5 German Mouse Clinic, Institute of Experimental Genetics, Helmholtz Zentrum München, German Research Center for Environmental Health, Neuherberg, Germany.; 6 Chair of Experimental Genetics, School of Life Science Weihenstephan, Technische Universität München, Freising, Germany.; 7 German Center for Diabetes Research (DZD), Neuherberg, Germany.; 8 Munich Cluster for Systems Neurology (SyNergy), Munich, Germany.; 9 Department of Neurology, Klinikum Passau, Passau, Germany.

**Keywords:** Neuroscience, Monogenic diseases, Neurological disorders, Sodium channels

## Abstract

Cortical spreading depression (CSD), a wave of depolarization followed by depression of cortical activity, is a pathophysiological process implicated in migraine with aura and various other brain pathologies, such as ischemic stroke and traumatic brain injury. To gain insight into the pathophysiology of CSD, we generated a mouse model for a severe monogenic subtype of migraine with aura, familial hemiplegic migraine type 3 (FHM3). FHM3 is caused by mutations in *SCN1A*, encoding the voltage-gated Na^+^ channel Na_V_1.1 predominantly expressed in inhibitory interneurons. Homozygous *Scn1a*^L1649Q^ knock-in mice died prematurely, whereas heterozygous mice had a normal lifespan. Heterozygous *Scn1a*^L1649Q^ knock-in mice compared with WT mice displayed a significantly enhanced susceptibility to CSD. We found L1649Q to cause a gain-of-function effect with an impaired Na^+^-channel inactivation and increased ramp Na^+^ currents leading to hyperactivity of fast-spiking inhibitory interneurons. Brain slice recordings using K^+^-sensitive electrodes revealed an increase in extracellular K^+^ in the early phase of CSD in heterozygous mice, likely representing the mechanistic link between interneuron hyperactivity and CSD initiation. The neuronal phenotype and premature death of homozygous *Scn1a*^L1649Q^ knock-in mice was partially rescued by GS967, a blocker of persistent Na^+^ currents. Collectively, our findings identify interneuron hyperactivity as a mechanism to trigger CSD.

## Graphical abstract

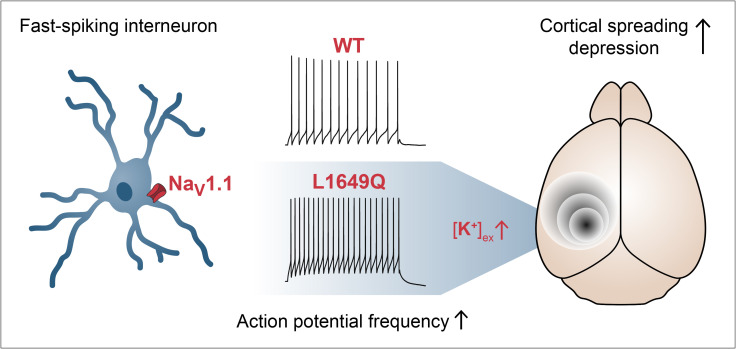

## Introduction

With a prevalence of 10% to 15% in the general population, migraine is one of the most common neurological diseases (1, 2) and is rated as one of the most frequent reasons for years lived with disability (3). About one-third of the patients suffer from additional transient neurological symptoms preceding the headache, named aura (2). The likely pathophysiological substrate of migraine aura is a wave of concomitant neuronal depolarization followed by inhibition of cortical activity slowly propagating across the cortex, termed cortical spreading depression (CSD) due to its discovery by EEG (4–7). The mechanisms underlying the initiation of CSD and the beginning of a migraine attack as well as the susceptibility to migraine and/or CSD remain elusive. This complicates the development of novel drugs for acute and prophylactic treatment.

Migraine is a multifactorial disease with a high heritability. Aside from disease-associated genetic variants in common migraine (8), there are rare monogenic forms of migraine with aura. Genetically engineered animal models of these monogenetic forms hold the potential to unravel the mechanisms not only of these rare genetic disorders, but also of CSD and migraine in general. Familial hemiplegic migraine (FHM) is an autosomal-dominant subtype of migraine with aura, characterized by severe auras with some degree of hemiparesis in addition to other neurological aura symptoms. Otherwise, the phenotype is remarkably similar to the common types of migraine. Genetically, FHM is heterogeneous, with 3 causative genes identified so far. The majority of FHM families carry missense mutations in either *CACNA1A* (FHM1) (9, 10), encoding a neuronal voltage-gated calcium channel (Ca_V_2.1), or *ATP1A2* (FHM2; refs. 11–13), encoding an ATP-dependent sodium-potassium pump expressed predominantly in astrocytes. Our group previously identified *SCN1A* as the third FHM gene (FHM3; ref. 14). *SCN1A* encodes the alpha subunit of a voltage-gated sodium channel (Na_V_1.1), which is mainly expressed and functionally important in inhibitory GABAergic interneurons (15–19). Of note, *SCN1A* has further been implicated in generalized epilepsy with febrile seizures plus (GEFS^+^; ref. 20) and Dravet syndrome (21), i.e., 2 monogenic epilepsy syndromes. While hundreds of missense or nonsense mutations have been identified in epilepsy (22), the mutational spectrum of FHM3 is limited, with a total of 10 missense mutations described so far (23).

Studies in transgenic mouse models for *Cacna1a* (24, 25) and *Atp1a2* (26, 27) have shown that mutations underlying FHM1 and FHM2 lead to a higher CSD susceptibility. This seems to be mediated by an increased concentration of glutamate in the extracellular space, causing hyperexcitability in the cerebral cortex (4, 28). The mechanism underlying FHM3 is still poorly understood. Previous studies have mostly been conducted in heterologous systems, with contradictory results. This is particularly true for mutation p.Leu1649Gln (L1649Q), for which different functional effects have been described. Kahlig et al. reported a markedly reduced surface expression of Na_V_1.1 and complete loss-of-function effect of L1649Q in tsA201 cells (29). In contrast, Cestèle et al. showed a gain-of-function of Na_V_1.1 expressed in tsA201 cells under different cell culture conditions and also in transfected neurons (30). A recent study reported an increased susceptibility for CSD in mice expressing another gain-of-function *Scn1a* mutation (p.Leu236Val; ref. 31). However, the mechanisms underlying CSD in these mice were not explored. To gain detailed insight into the pathophysiology of FHM3, we used a homologous recombination approach to generate a knock-in mouse model for the human FHM3 mutation L1649Q and analyzed the molecular, cellular and network mechanisms in these animals with a combination of in vitro and in vivo techniques.

## Results

### Generation of Scn1a^L1649Q^ knock-in mice.

**Figure 1:**
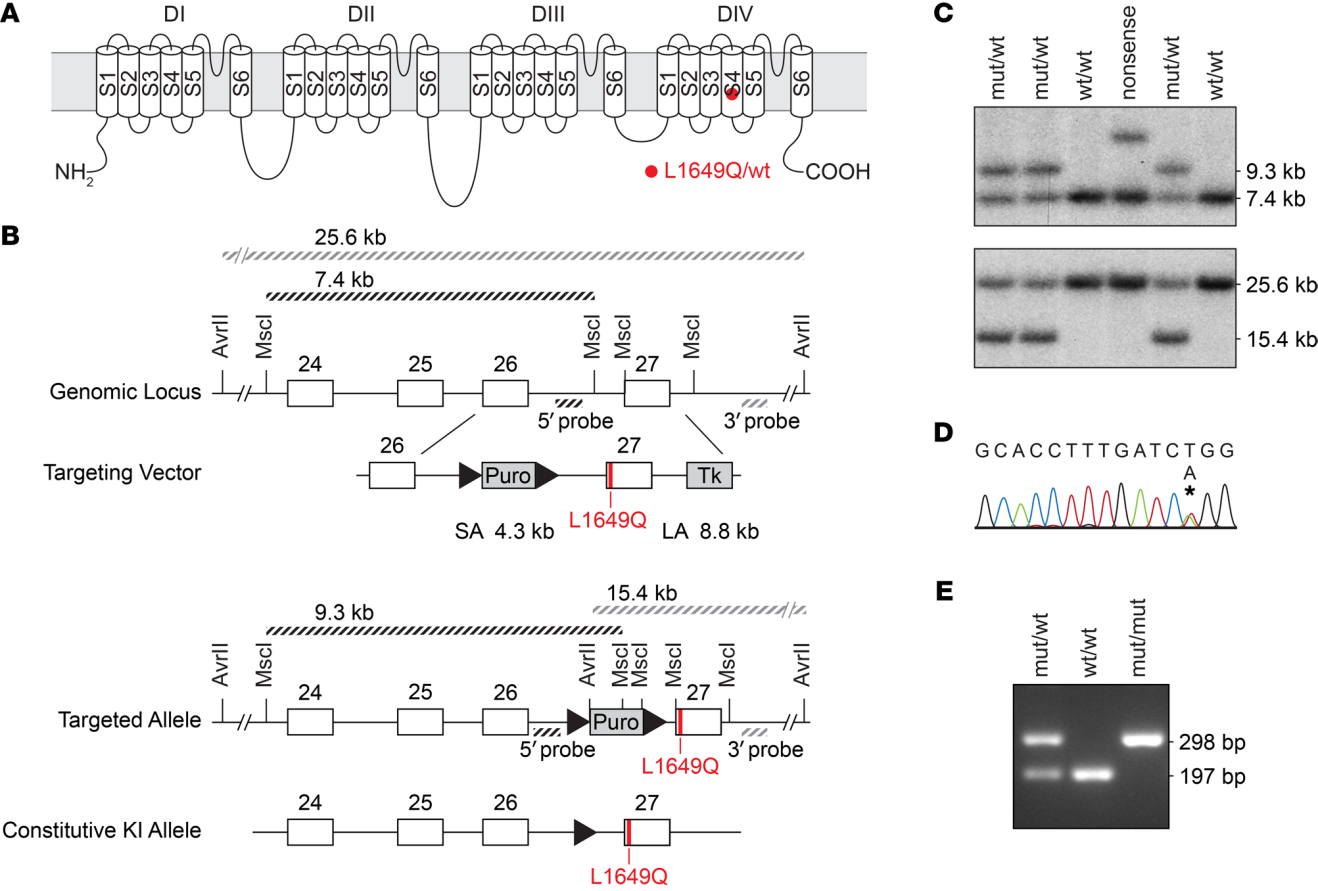
Generation of *Scn1a*^L1649Q^ knock-in mice. (**A**) The mutation p.Leu1649Gln (L1649Q) is located in segment 4 (S4) of domain IV of the voltage gated sodium channel Na_V_1.1. (**B**) Scheme of important parts of the *Scn1a* WT allele, the targeting vector, and the mutated allele after homologous recombination. White boxes indicate exons; triangles indicate F3 sites. Restriction sites of MscI and AvrII are depicted; the probes used for Southern blot and the length of the individual restriction fragments after digestion of genomic DNA are indicated in the scheme. (**C**) Proof of homologous recombination of 5′-side (upper blot) and 3′-side (lower blot) by Southern blot analysis. For Southern blot analysis, MscI (5′-side) and AvrII (3′-side) digested genomic DNA of mutant and WT mice were used. (**D**) Sequencing trail showing correct insertion of c.4946T>A (asterisk), predicting L1649Q on the protein level. (**E**) Genotyping of *Scn1a*^L1649Q^ offspring by PCR analysis of genomic DNA.

The human *SCN1A* L1649Q point mutation is located in segment 4 of domain IV of the voltage-gated sodium channel Na_V_1.1 (Figure 1A). To introduce L1649Q into the ortholog mouse *Scn1a* gene at the corresponding position (exon 27 of the mouse *Scn1a* gene, p.Leu1649Gln), we used a homologous recombination approach (Figure 1B). The correct homologous recombination and the presence of the point mutation was confirmed by Southern blot analysis of genomic DNA (Figure 1C) and Sanger sequencing (Figure 1D). Transgenic animals were backcrossed with C57Bl/6N females for more than 6 generations. The offspring were genotyped using PCR (Figure 1E).

The median litter size in heterozygous/heterozygous interbreeding of *Scn1a*^L1649Q^ knock-in animals was 7 (IQR 3, data not shown) and did not differ from litter sizes of heterozygous/WT interbreeding. The observed distribution of genotypes did not differ from the expected Mendelian distribution (χ^2^=0.041, *P =* 0.98, χ^2^ test; data not shown).

### Premature death of homozygous Scn1a^L1649Q^ knock-in mice.

**Figure 2:**
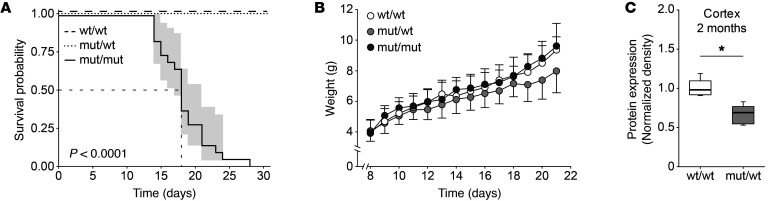
Premature death of homozygous *Scn1a*^L1649Q^ knock-in mice. (**A**) Kaplan-Meier plot from *Scn1a*^L1649Q^ knock-in offspring. Homozygous offspring showed significantly reduced survival (median survival was estimated at 18 days (95% CI: 16–21 days, *P <* 0.0001; group sizes: wt/wt, *n =* 28; mut/wt, *n =* 50; mut/mut, *n =* 22; log rank test). (**B**) There was no difference in bodyweight gain after birth in *Scn1a*^L1649Q^ knock-in offspring among different genotypes (the maximum number of animals per group is indicated as group size: wt/wt, *n =* 34; mut/wt, *n =* 56; mut/mut, *n =* 24; 1-way ANOVA), data are shown as mean ± SD. (**C**) Na_V_1.1 expression level normalized to actin levels of cortical tissue prepared from WT and heterozygous *Scn1a*^L1649Q^ knock-in littermates at the age of 2 months. Western blot was performed using Na_V_1.1 and β-actin antibodies. The Na_V_1.1 protein level in cortex samples of heterozygous *Scn1a*^L1649Q^ knock-in mice was significantly reduced compared with samples of WT littermates (*P < 0.05, group sizes: wt/wt, *n =* 5; mut/wt, *n =* 5; Mann-Whitney rank sum test).

Homozygous *Scn1a*^L1649Q^ offspring had a substantially reduced lifespan, with a median survival at postnatal day 18 (95% CI: 16–21 days). By contrast, heterozygous littermates were fully viable, and their survival was identical to WT littermates (Figure 2A). Heterozygous and homozygous knock-in animals could not be distinguished from WT littermates with respect to behavior, appearance, and biometric parameters, including body weight, for the first 2 postnatal weeks (Figure 2B). Homozygous animals displayed rare hemiplegic attacks with circling behavior as previously described in FHM1 knock-in animals (25). A systematic screen with comprehensive phenotyping revealed no significant differences between heterozygous *Scn1a*^L1649Q^ knock-in mice and WT littermates (Supplemental Table 1; supplemental material available online with this article; https://doi.org/10.1172/JCI142202DS1), except for a shorter QT interval duration (*P =* 0.003) and a reduced QTc dispersion (*P =* 0.006) in female heterozygous *Scn1a*^L1649Q^ knock-in mice (Supplemental Tables 2 and 3). However, this phenotype was not observed in homozygous mice shortly before their premature death (Supplemental Figure 1). Detailed results of the phenotypic screen are available online at the German Mouse Clinic phenomap website (www.mouseclinic.de).

### mRNA and protein expression of Scn1a in knock-in animals.

Quantitative PCR analysis of *Scn1a* mRNA expression levels revealed no significant differences between genotypes in cortex and brainstem (Supplemental Figure 2A). Likewise, Na_V_1.1 protein expression levels did not differ between genotypes in P16 animals (Supplemental Figure 2B). By contrast, cortical Na_V_1.1 levels in 2-month-old mice (Figure 2C) were significantly lower in heterozygous knock-in animals (*P <* 0.05). A similar trend was noted in the brainstem, however without reaching statistical significance. Due to premature death of homozygous animals, this analysis was limited to WT and heterozygous mice. mRNA expression levels of other sodium channels (*Scn2a*, *Scn3a*, and *Scn8a*) were largely similar between genotypes (Supplemental Figure 3).

### Slowed inactivation of mutant Na_V_1.1 channels in acutely dissociated neurons.

**Figure 3:**
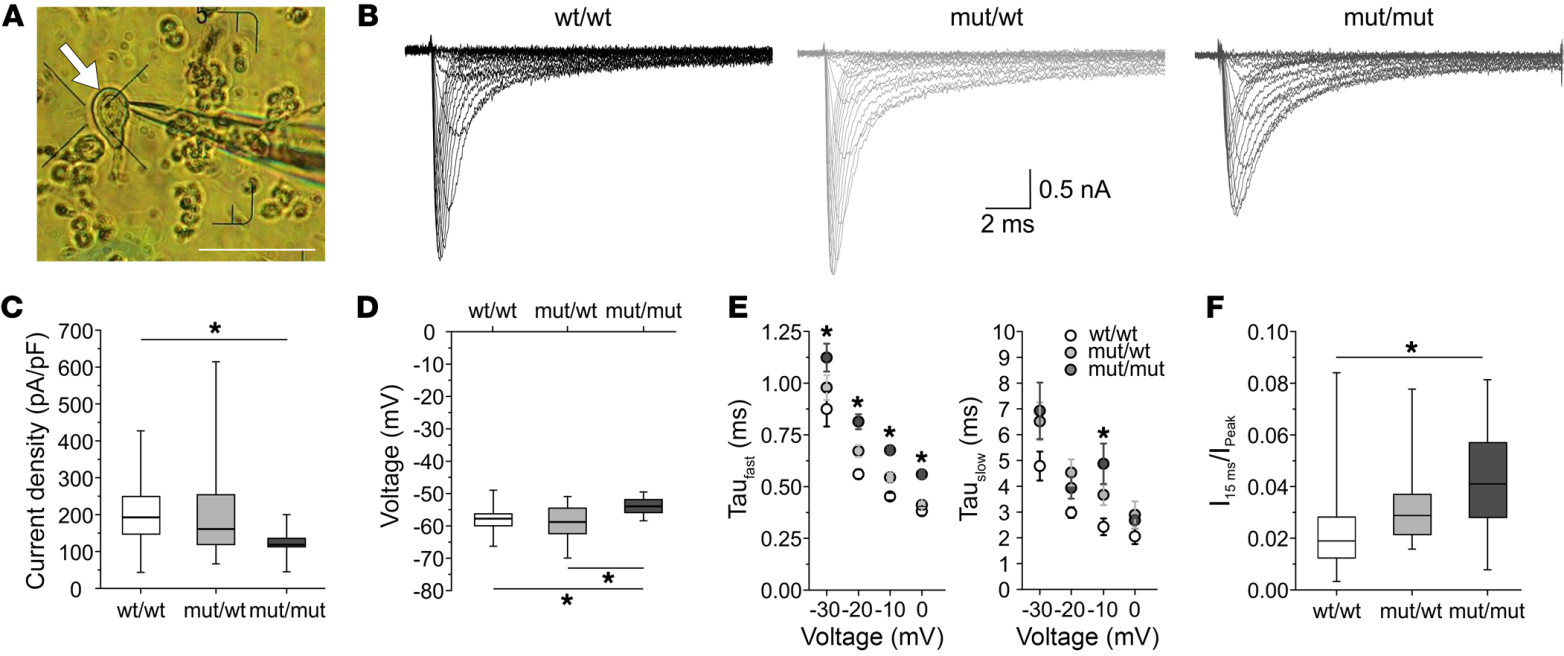
Slowed inactivation of Na^+^ currents in acutely isolated cerebellar Purkinje cells of mutant mice. (**A**) Image of an acutely dissociated cerebellar Purkinje neuron (marked by an arrow) and a patch pipette. Scale bar: 100 μm. (**B**) Representative traces of Na^+^ currents recorded from dissociated neurons of wt/wt, mut/wt, and mut/mut animals were elicited by voltage steps from –70 to 5 mV in 5 mV increments (duration 15 ms, holding potential –90 mV). (**C**) Box plots of the peak current density of neurons recorded from animals with the indicated phenotypes (*P < 0.05, ANOVA on ranks with Dunn’s post hoc test, wt/wt: *n =* 27; mut/wt: *n =* 33, mut/mut: *n =* 12). (**D**) Box plots of the voltage of half-maximal inactivation for the indicated phenotypes. V_1/2_ was significantly shifted to more depolarized potentials in neurons of mut/mut animals in comparison to wt/wt and mut/wt (wt/wt: *n =* 27; mut/wt: *n =* 33, mut/mut: *n =* 12, *P < 0.05 ANOVA on ranks with Dunn’s post hoc test). (**E**) Dot plots of fast (left) and slow (right) time constants of fast inactivation, respectively, plotted over different voltage steps. Data are shown as mean ± SEM (wt/wt: *n ≤* 14; mut/wt: *n ≤* 31, mut/mut: *n ≤* 13, *P < 0.05 2-way ANOVA with Tukey test for pairwise multiple comparison). (**F**) Box plots of remaining current at the end of a 15 ms test pulse to –25 mV (I_15ms_) divided by the peak current (I_Peak_) recorded at the same voltage. The remaining current was significantly increased in neurons of mut/mut animals in comparison to wt/wt and mut/wt (wt/wt: *n =* 20; mut/wt: *n =* 25, mut/mut: *n =* 9, *P < 0.05 ANOVA on ranks with Dunn’s post hoc test).

Na_V_1.1 is one of the primary Na^+^ channels expressed in Purkinje cells (32), which can be easily identified based on their size and typical shape (Figure 3A). To determine the functional effects of the L1649Q mutation, we first performed whole-cell patch clamping of acutely isolated Purkinje cells from WT, hetero-, and homozygous mice between P19 and P25, which enabled recordings of large Na^+^ currents without space clamp artefacts (Figure 3B). Current density was similar in Purkinje cells dissociated from WT and heterozygous mice but significantly reduced in homozygous mice (Figure 3C). However, current density was rather variable for all genotypes and less data could be obtained for homozygous mice because of their reduced survival. The activation properties of the Na^+^ currents were similar between genotypes (See Supplemental Table 4).

Several properties of the inactivation process were significantly changed. The voltage of half-maximal steady-state inactivation, while similar between WT and heterozygous mice, was shifted by +4 mV in homozygous mice (Figure 3D). The time course of inactivation was described as a sum of 2 exponential functions (see Supplemental Methods). Both time constants were larger for heterozygous versus WT mice, and for homozygous versus both WT and heterozygous mice (Figure 3E). As a consequence of the slowed inactivation, the remaining Na^+^ current, measured at –25 mV at the end of a 15 ms pulse relative to the peak current (I_15ms_/I_peak_), was significantly elevated in homozygous mice (Figure 3F). The time course of recovery from inactivation was not different between mutant and WT mice (data not shown).

### Gain-of-function effect of L1649Q resulted in increased firing of interneurons in acute brain slices.

**Figure 4:**
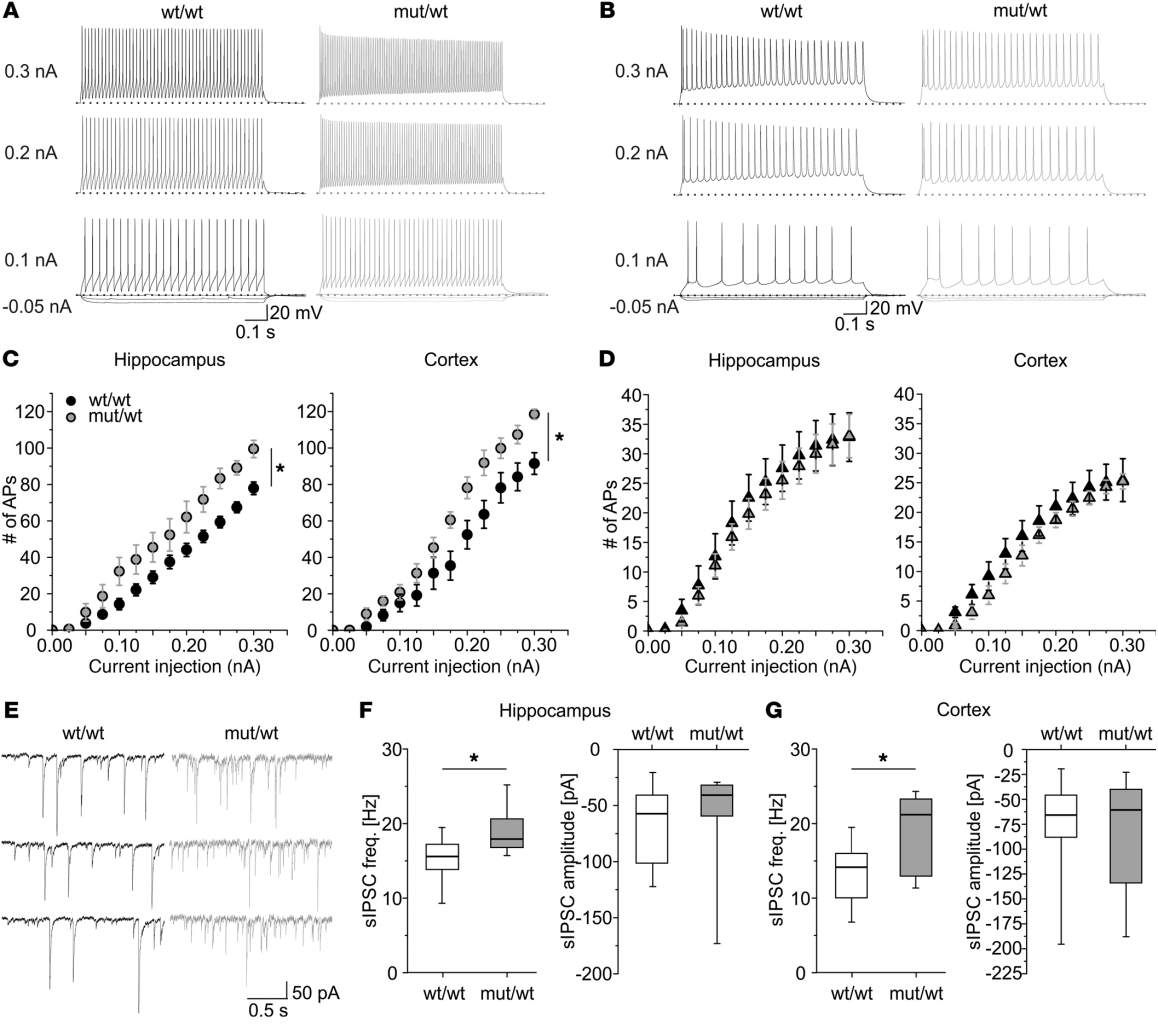
Hyperexcitability of GABAergic neurons and increased GABAergic transmission in acute slices of heterozygous *Scn1a*^L1649Q^ knock-in mice. Representative whole-cell current clamp recordings of action potential series in hippocampal GABAergic (**A**) and layer 5 pyramidal neurons (**B**) of wt/wt (left) and mut/wt (right) animals. Voltage traces upon injection of –0.05, –0.025, 0, and 0.1 nA; middle: 0.2 nA, bottom: 0.3 nA. Dashed lines show holding potential of –70 mV. Number of APs per trace plotted versus size of current injection for fast-spiking inhibitory neurons (**C**) of hippocampal CA1 region (left) and cortical layer 4 (right) as well as pyramidal neurons (**D**) located in the hippocampal CA1 region (left) and cortical layer 5 (right). The area under the curve was significantly increased only for inhibitory neurons of heterozygous animals compared with WT littermates, indicating a higher frequency of AP firing (*P < 0.05; Mann-Whitney rank sum test). CA1: inhibitory neurons: wt/wt: *n =* 18 cells from 6 animals (18/6); mut/wt: 10/6; pyramidal cells: wt/wt: 11/5; mut/wt: 12/6. Cortex: inhibitory neurons: wt/wt: 7/4; mut/wt: 6/5; pyramidal cells: wt/wt: 9/5; mut/wt: 13/6. Data are shown as mean ± SEM. (**E**) Representative whole-cell current traces showing recorded sIPSCs from hippocampal pyramidal neurons within the CA1 region recorded in slices of wt/wt (left) or mut/wt (right) animals. Membrane voltage was clamped to –70 mV. The sIPSC frequency as recorded in (**E**) was significantly increased for mut/wt animals in both cortical (**F**, left) and hippocampal (**G**, left) pyramidal cells, whereas the sIPSC amplitude did not change (**F **and **G**, right; *P < 0.05; Mann-Whitney rank sum test). Hippocampus: wt/wt: *n =* 8 cells from 3 animals (8/3); mut/wt: 7/3. Cortex: wt/wt: 19/6; mut/wt: 10/3.

To further study the functional effect of the L1649Q mutation, we moved to electrophysiological analyses in acute brain slices from heterozygous animals versus WT littermates. We used thalamocortical and horizontal hippocampal slices from postnatal day 14–20 to perform whole-cell patch clamp recordings of inhibitory and excitatory neurons within cortex or hippocampus as previously described (17). In both the cortex and the hippocampal CA1 region, input-output curves revealed a significantly increased action potential frequency in fast-spiking inhibitory interneurons of heterozygous animals (i.e., in the cell type in which Na_V_1.1 is mainly expressed) (Figure 4, A and C). Recordings in cortical layer 5 and CA1 pyramidal neurons did not reveal differences between heterozygous and WT animals (Figure 4, B and D and Supplemental Table 5). No significant differences were found for cortical and hippocampal regular spiking inhibitory interneurons (see Supplemental Figure 4).

To characterize alterations of inhibitory input, we studied spontaneous and miniature inhibitory postsynaptic currents (IPSCs) from pyramidal cells in both cortex and hippocampus. As expected for a gain-of-function Na^+^-channel mutation increasing neuronal firing in interneurons, we observed a significantly increased frequency of spontaneous IPSCs (sIPSCs) in heterozygous animals versus WT littermates (Figure 4, E–G). Miniature IPSCs (mIPSCs), recorded by applying 1 μM tetrodotoxin (TTX) to block action potentials, did not reveal a difference between WT and heterozygous animals (data not shown).

**Figure 5:**
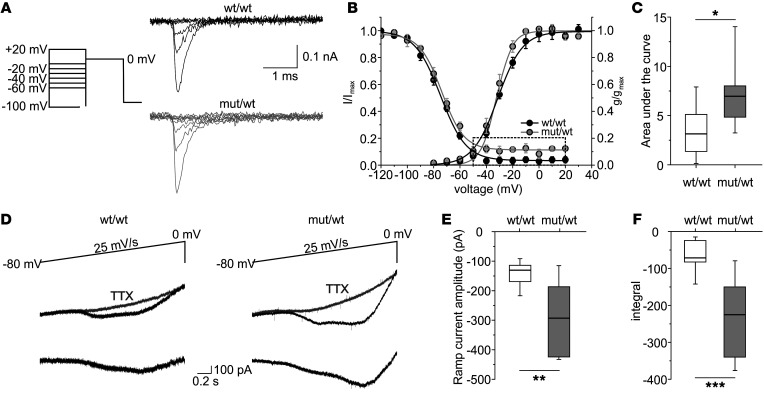
Increased ramp Na^+^ currents of hippocampal inhibitory neurons in acute brain slices. (**A**) Recordings of steady-state fast inactivation of Na^+^ currents from nucleated patches of inhibitory neurons of wt/wt (top) and mut/wt (bottom) animals. Steady-state inactivation induced by 30 ms–lasting conditioning pulses to different potentials (between –120 mV and 20 mV in 10 mV increments) and 30 ms test pulse to 0 mV (holding potential –90 mV). Shown are currents elicited by test pulse to 0 mV (conditioning pulses of –100 mV, –60 mV, –50 mV, –40 mV, –30 mV, –20 mV, –10 mV shown on the left). (**B**) Mean voltage-dependence of steady-state Na^+^-channel activation and fast inactivation (± SEM). Lines represent Boltzmann functions fit. V_1/2_ and k_V_ slope factor for activation and inactivation curves were not different between genotypes. Dashed box represents the part used for analyzing area under the curve (AUC) shown in **C**. (**C**) Box plot of values of AUC of steady-state fast inactivation between –50 mV and 20 mV, indicating a persistent, steady-state Na^+^ current, which was significantly increased in inhibitory neurons of mut/wt compared with wt/wt animals (*P =* 0.029; Mann-Whitney rank sum test; wt/wt, *n =* 8; mut/wt, *n =* 8). (**D**) Representative whole-cell recordings elicited by voltage ramps from –80mV to 0 mV showing ramp Na^+^ currents of fast-spiking neurons, which were blocked by TTX. Bottom traces show net ramp current after subtracting traces recorded with TTX from those without. (**E**) Box plots of ramp current amplitudes recorded in inhibitory neurons from wt/wt and mut/wt animals. The ramp current was significantly increased in neurons of mut/wt animals compared with wt/wt (**P < 0.01; Mann-Whitney rank sum test; wt/wt: *n =* 9 cells from 2 animals [9/2]; mut/wt: 7/2). (**F**) Box plots of integral of recorded ramp currents, which were significantly increased in neurons of mut/wt animals compared with wt/wt (***P < 0.001; Mann-Whitney rank sum test; wt/wt: 9/2; mut/wt: 7/2).

To dissect the alterations of Na_V_1.1 function causing this hyperexcitability of inhibitory neurons in more detail, we used nucleated patch recordings, allowing adequate voltage control from identified neurons (33). Nucleated patches from CA1 inhibitory neurons at the border between *stratum oriens* and *stratum pyramidale* had Na^+^-current amplitudes of 100 to 400 pA without showing any differences in current amplitudes (wt/wt: –294.3 ± 46.6 pA; mut/wt: –206.8 ± 27.0 pA) (Figure 5A). Half-maximal voltages and slopes of steady-state activation and inactivation curves were similar for heterozygous and WT animals (Figure 5B). However, the inactivation curve showed a 3-fold larger baseline and thus an increase in the area under the curve between –50 mV and +20 mV (Figure 5C), hinting at an increased persistent or steady-state Na^+^ current, as it was detected before for the L1649Q mutation in transfected neurons (30). Presumably due to the small currents and a high variability, we were not able to detect a significantly increased current at the end of the test pulses in nucleated patches (see Supplemental Table 6). To assess a persistent current in neurons in their native environment, we performed whole-cell recordings using slow voltage ramps of hippocampal GABAergic neurons in the CA1 region at the border between *stratum oriens* and *stratum pyramidale*. We found a significant increase in the Na^+^ current elicited by voltage ramps for heterozygous compared with WT animals, as revealed by using TTX as specific Na^+^ channel blocker (Figure 5, D–F).

### Higher susceptibility to CSD in vivo.

**Figure 6:**
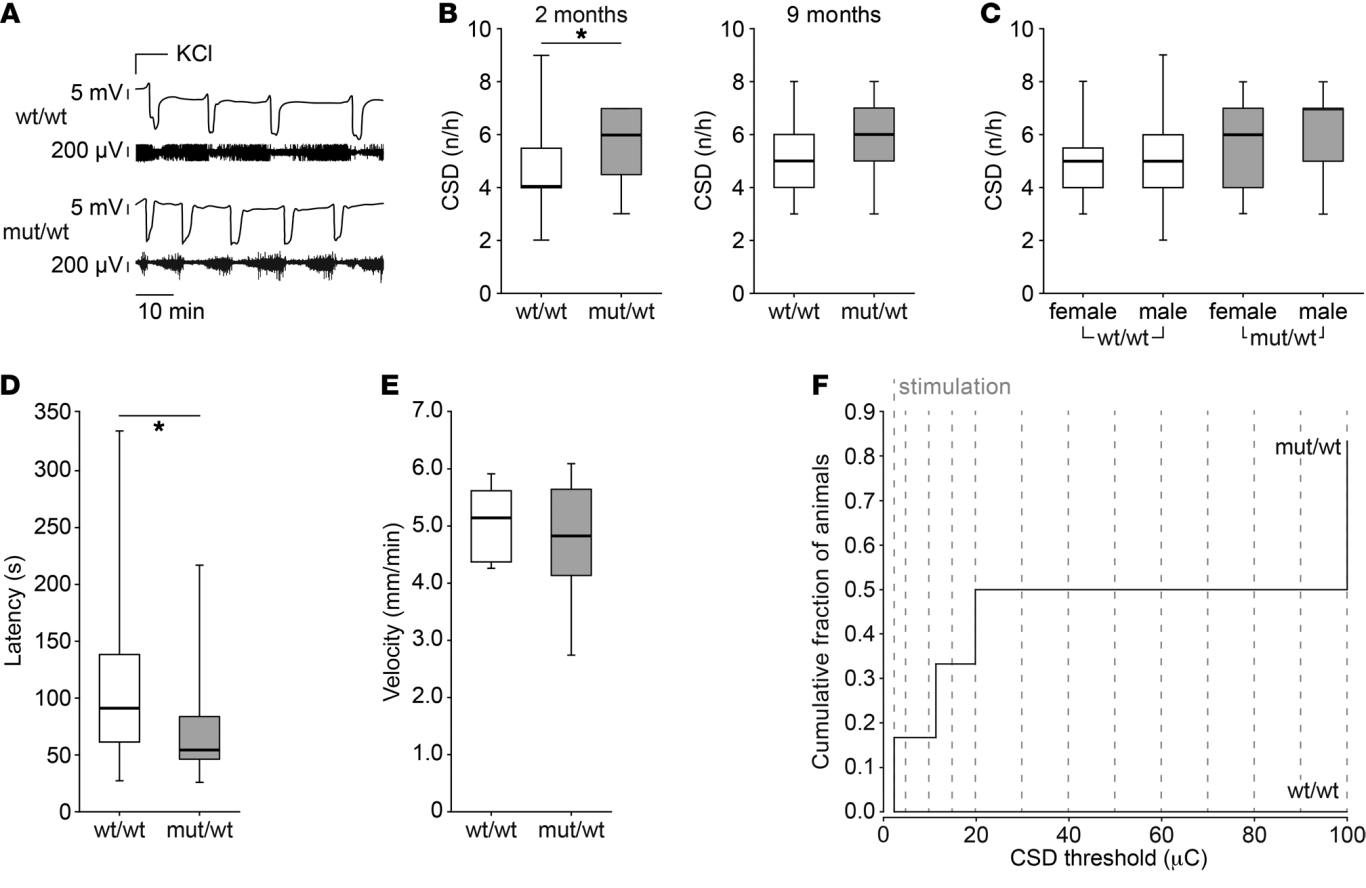
Higher susceptibility to CSD in heterozygous animals in vivo. (**A**) Representative recordings of DC potential (top) and ECoG signal (below) of CSD in WT and heterozygous littermates after local application of 300 mM KCl. (**B**) Box plots represent the frequency of CSD events, elicited as in **A**, showing a higher CSD frequency in heterozygous animals compared with WT littermates in 2-month-old animals (*P < 0.05) and a trend in 9-month-old animals (*P <* 0.10) (group sizes: 2 months: wt/wt, *n =* 17; mut/wt, *n =* 13; 9 months: wt/wt, *n =* 18; mut/wt, *n =* 19; Mann-Whitney rank sum test). (**C**) CSD frequency in WT and heterozygous male and female animals without differences between sexual phenotype (*P =* 0.67; group sizes: female: wt/wt, *n =* 17; mut/wt, *n =* 13; male: wt/wt, *n =* 18; mut/wt, *n =* 19; Mann-Whitney rank sum test). (**D**) Latency (s) of the first CSD after stimulation. Heterozygous littermates showed a shorter latency (*P < 0.05; group size: wt/wt, *n =* 16; mut/wt, *n =* 13; Mann-Whitney rank sum test). (**E**) CSD propagation velocity (mm/min) was similar in both genotypes (*P =* 0.488; group size: wt/wt, *n =* 12; mut/wt, *n =* 13; Mann-Whitney rank sum test). (**F**) CSD threshold determined by electrical stimulation. Five of 6 heterozygous animals (83%) showed a CSD at the given thresholds, whereas these stimulation intensities were not able to elicit any CSDs in WT littermates (median: 20 μC; group size: wt/wt, *n =* 6; mut/wt, *n =* 6).

Having determined the molecular and cellular effects of the L1649Q mutation, we examined the consequences on migraine-relevant parameters in intact animals, eliciting CSD by either application of 300 mM KCl or electrical stimulation. Figure 6A illustrates representative recordings of direct current (DC) potential and electrocorticogram signal of CSD events in WT and heterozygous littermates which were experimentally induced by local application of 300 mM KCl. Quantitative analysis of CSD events revealed a significantly increased frequency in heterozygous animals versus WT littermates at 2 months of age (*P <* 0.05, wt/wt: median 4 [IQR 1.5], mut/wt: median 6, [IQR 2.5]), whereas only a trend was observed in older (9 months) animals (*P =* 0.092; wt/wt: 5 [IQR 2], mut/wt 6 [IQR 2]) (Figure 6B). This effect was found to be stable irrespective of sex (*P =* 0.488; wt/wt: 5 [1.5] female, 5 [2] male; mut/wt: 6 [3] female, 7 [2] male) (Figure 6C). In addition, the latency from KCl application to the onset of CSD was significantly reduced in heterozygous animals (*P <* 0.05, wt/wt: 91 [77], mut/wt: 54 [38]), indicating a higher CSD susceptibility (Figure 6D), but CSD propagation velocity was similar in both groups (*P =* 0.67; wt/wt: 5.05 [1.2], mut/wt 4.76 [1.3]) (Figure 6E). To determine the threshold for eliciting CSD, we used electrical stimulation with increasing intensity. In 6 WT animals, we were not able to elicit a CSD even with the highest stimulation (100 μC), whereas in 5 of 6 heterozygous animals CSD could be generated in a range from 3 to 100 μC, indicating a substantially lowered threshold in mutant animals (Figure 6F).

### Increase in extracellular potassium levels in acute brain slices of heterozygous knock-in mice during elicited CSD.

**Figure 7:**
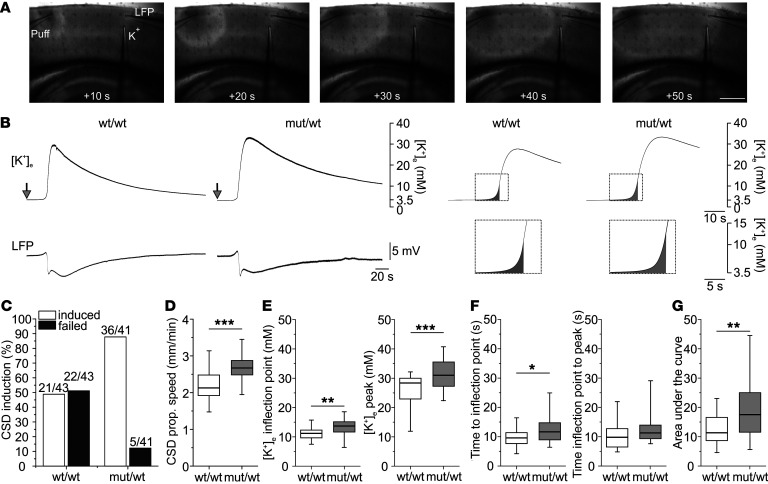
Increased extracellular [K^+^]_e_ during CSD in slices of heterozygous versus WT animals. (**A**) IOS of CSD induced by local KCl application (Puff) in slice of wt/wt animal with K^+^ sensitive and LFP electrodes. Scale bar: 500 μm. (**B**) Traces of K^+^ sensitive recording (top) and LFP signal (bottom) during CSD for slices of wt/wt (left) and mut/wt animals (right). CSD-recording elicited by KCl-application (arrow) (left). Averaged [K^+^]_e_ dynamics before and during CSD (wt/wt: 21 slices; mut/wt: 30 slices) (right). Magnified boxes indicate AUC from point at which K^+^ signal left baseline (threshold: +0.1 mM) to inflection-point of [K^+^]_e_ curve (see Supplemental Figure 5B). (**C**) Success rate of CSD induction in slices of both genotypes (wt/wt: *n =* 43 slices from 12 animals [43/12]; mut/wt: 41/11; *P <* 0.001; Fisher’s exact test). CSD failed category includes CSDs that aborted before reaching K^+^ and DC electrodes. (**D**) Propagation velocity was increased in mut/wt compared with wt/wt animals (wt/wt: 22/10; mut/wt: 31/11; ***P < 0.001; Mann-Whitney rank sum test). (**E**) [K^+^]_e_ at inflection point of K^+^ signal (determined as in Supplemental Figure 5B) and maximum [K^+^]_e_ (right) during CSD. [K^+^]_e_ was increased in slices of heterozygous compared with WT animals (wt/wt: 22/10; mut/wt: 31/11; **P < 0.01, ***P < 0.001; Mann-Whitney rank sum test). (**F**) [K^+^]_e_ increased earlier in slices of heterozygous compared with WT animals. Time at which [K^+^]_e_ started to increase to inflection point of K^+^ signal (left) and from inflection point to maximum peak (right). Initial rise of [K^+^]_e_ started earlier in mut/wt compared with wt/wt animals (wt/wt: 22/10; mut/wt: 31/11; *P < 0.05; Mann-Whitney rank sum test). Time from inflection point to peak concentration was not different between genotypes (wt/wt: 22/10; mut/wt: 31/11; Mann-Whitney rank sum test). (**G**) AUC from start of [K^+^]_e_ increase to inflection point of K^+^ signal (as shown in **B**) increased in slices of mut/wt compared with wt/wt animals (wt/wt: 22/10; mut/wt: 31/11; **P < 0.01; Mann-Whitney rank sum test).

We aimed to unravel the mechanism by which an increased ramp Na^+^ current can cause a higher susceptibility and lower the threshold for CSD. Cestèle et al. (34) had already suggested that a higher activity of interneurons caused by an increased persistent Na^+^ current may raise the extracellular K^+^ concentration ([K^+^]_e_). To further explore this hypothesis, we used acute cortical brain slices in a setup combining an extracellular voltage electrode, a K^+^-selective electrode and intrinsic optical signal (IOS) imaging to capture the CSD (Figure 7A). CSD was elicited by a 200 mM KCl puff application lasting 400 ms, at least 1000 μm distant from the recording site. A successful induction of a CSD with a greater than 900 μm propagating wave in the IOS images, a DC shift in the local field potential (LFP) recordings, and an increase in [K^+^]_e_ was elicited in 87.8% of slices of heterozygous animals, whereas CSD was successfully induced in only 48.8% of slices of WT littermates (*P <* 0.001; Fisher’s exact test; Figure 7C). In this setting, we found that the CSD propagation velocity in slices of heterozygous animals was slightly increased compared with WT littermates (Figure 7D). The [K^+^]_e_ at the inflection point, indicating the maximum speed of [K^+^]_e_ calculated by the first derivative (see Supplemental Figure 5B), and the maximum [K^+^]_e_ of the whole K^+^ recording were significantly increased in slices of heterozygous compared with WT animals (Figure 7, B and E). To further investigate the K^+^ accumulation, we defined the beginning of the extracellular [K^+^]_e_ increase as a +0.1 mM change of baseline concentration. The time between the beginning and the inflection point of the [K^+^]_e_ curve was longer in heterozygous animals, whereas the time from the inflection point to the maximum [K^+^]_e_ as well as the rise time and decay of the [K^+^]_e_ measurement were not different between slices of both phenotypes, indicating an earlier start of the [K^+^]_e_ increase in heterozygous animals (Figure 7F and Supplemental Figure 6). In addition, area under the curve analysis confirmed an increased potassium shift within this early interval (Figure 7B, right, and G).

### Effects of the Na^+^-current blocker GS967 in vitro and in vivo.

**Figure 8:**
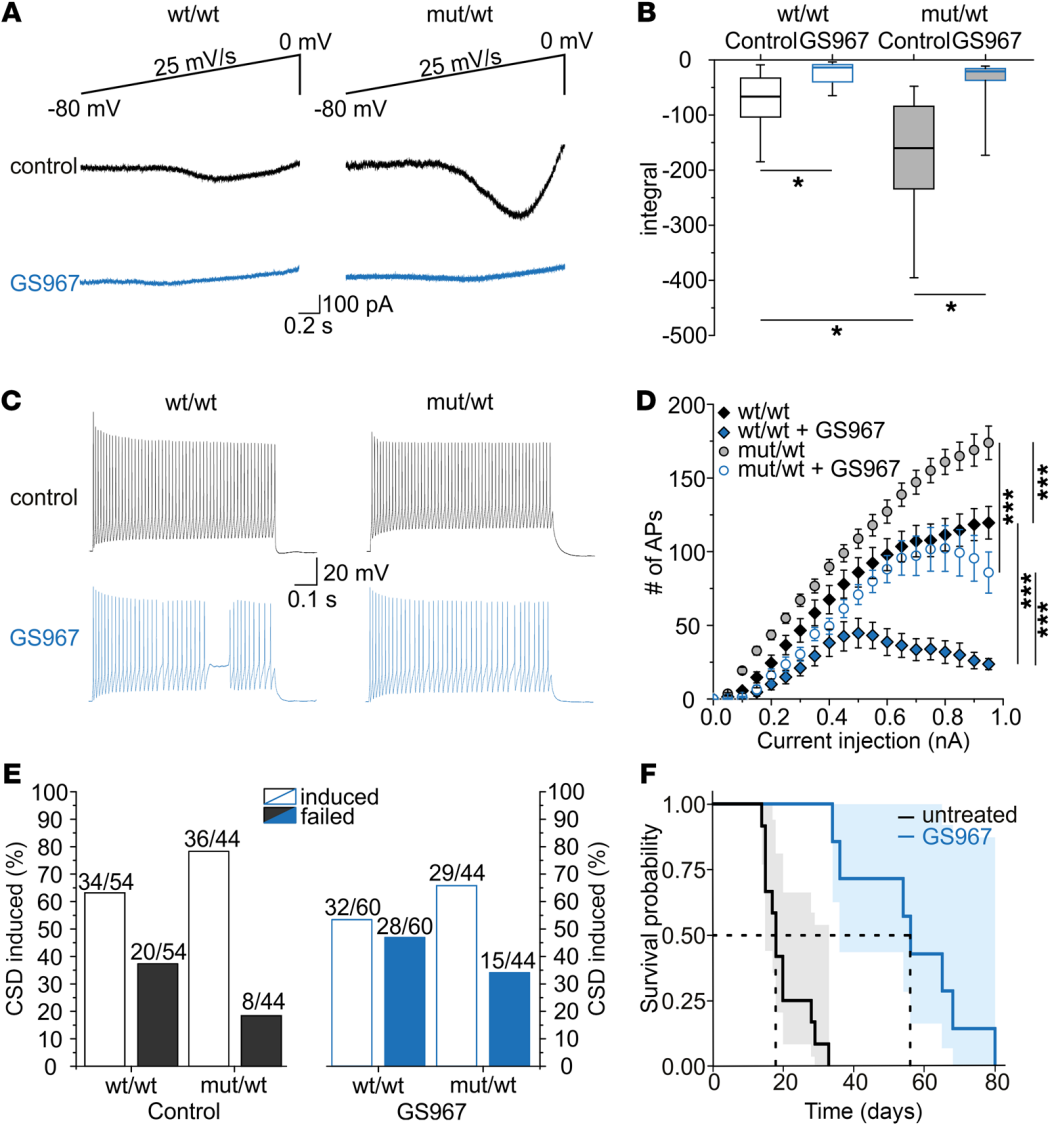
The late Na^+^-current blocker GS967 blocks Na^+^ ramp currents, reduces number of action potentials, and prolongs the lifespan of homozygous animals. (**A**) Representative net persistent ramp Na^+^ currents of hippocampal fast-spiking neurons, as shown in Figure 5D, after subtracting the traces recorded with TTX from those without TTX. Black traces show recordings before application of 3 μM GS967, blue traces show recordings from the same neurons after GS967 application. (**B**) Box plots of the integral of TTX-subtracted ramp currents with and without GS967 for wt/wt and mut/wt animals. The integral was reduced by application of GS967 in neurons of wt/wt and mut/wt animals (*P < 0.05; 2-way ANOVA; wt/wt: *n =* 12 cells from 3 animals [12/3]; mut/wt: 12/3). (**C**) Representative voltage traces of hippocampal CA1 inhibitory neurons recorded in slices of wt/wt (left) and mut/wt (right) animals at a current injection of 0.3 nA without (top) and with (bottom) application of 3 μM GS967. (**D**) Number of APs per trace plotted versus size of current injection for fast-spiking inhibitory neurons of hippocampal CA1 region with and without GS967 application. The AUC for all current injections (shown in plot) as well as up to 0.3 nA was significantly decreased by GS967 administration in WT and heterozygous animals (***P < 0.001, 2-way ANOVA; wt/wt: *n =* 15 cells from 5 animals [15/5], mut/wt: 16/5. 2-way ANOVA). Data are shown as mean ± SEM. (**E**) Success rate of CSD induction in slices of both genotypes without (control) and with GS967 application (wt/wt control: *n =* 54 slices from 28 animals [54/28], mut/wt control: 44/20; *P < 0.05, χ^2^ test; wt/wt GS967: 60/28; mut/wt GS967: 44/20; *P =* 0.94; χ^2^ test). (**F**) Kaplan-Meier plot of homozygous *Scn1a*^L1649Q^ knock-in mice treated with GS967 (median survival: 56 days) and untreated homozygous control mice (median survival: 18 days) (*P <* 0.001; group size: untreated: *n =* 12, treated: *n =* 7; log rank test).

The late Na^+^-current blocker GS967 (35) decreased the Na^+^ current elicited by voltage ramps in GABAergic interneurons (Figure 8, A and B) using the same methods as described before (Figure 5, E and F). Additionally, we studied the effect of GS967 at a wide range of action potential frequencies using current injections up to 0.95 nA. The administration of GS967 reduced the number of action potentials in fast-spiking interneurons of heterozygous and WT animals with a more pronounced effect for high frequencies, which was due to a termination of firing with a depolarization block, most probably caused by the use-dependent block of Na^+^ channels by GS967 administration (ref. 36 and Figure 8, C and D).

Whereas more CSDs were elicited in slices of heterozygous animals in the absence of GS967, the induction rate for CSDs was not different between GS967-perfused slices of WT and heterozygous littermates (Figure 8E). As a proof-of-concept for in vivo pharmacological intervention, we tested a beneficial effect of GS967 on the lifespan of homozygous *Scn1a*^L1649Q^ mice. Applying GS967 at a dose that has no adverse effects on WT mice (37) substantially increased the survival of homozygous *Scn1a*^L1649Q^ mice (Figure 8F) from a median survival of 18 days in untreated animals to 56 days in treated animals (*P <* 0.001).

## Discussion

We here present the generation and in-depth characterization of what we believe is a novel knock-in FHM3 mouse model. Our study reveals a mechanism to elicit CSD by increased interneuron activity due to a gain-of-function *SCN1A* mutation. Our results demonstrate a clear-cut FHM phenotype, represented by an increased susceptibility to CSD shown in electrophysiological in vitro and in vivo studies. The underlying mechanisms were elucidated by recordings in dissociated neurons and acute brain slices. We identified a cascade from a slowed and incomplete Na^+^-channel inactivation to a hyperactivity of cortical and hippocampal fast-spiking inhibitory interneurons and increased [K^+^]_e_ during CSD recordings. A specific blocker of persistent Na^+^ currents was able to alleviate the CSD susceptibility in slices of heterozygous animals and to prolong the lifespan of homozygous animals. Our study expands previous concepts of CSD pathophysiology in FHM, which so far mainly focused on enhanced glutamatergic neurotransmission. While obtained in a genetic model of migraine with aura, our findings might also be generalizable to CSD in the context of other neurological diseases such as traumatic brain injury, ischemic stroke, and cerebral hemorrhage (38).

### The pathophysiological cascade of the L1649Q mutation.

Our data from acute brain slices and Purkinje cells clearly reveal a gain-of-function effect of the L1649Q mutation, demonstrated by substantially increased action potential firing rates in fast-spiking inhibitory neurons located in cortical layer 4 or the hippocampal CA1 region, but not in excitatory pyramidal cells. Consequently, recordings of sIPSCs in pyramidal neurons in layer 5 and the hippocampal CA1 region display an increased GABAergic synaptic activity.

The observed hyperexcitability of inhibitory interneurons does not fit to previously established pathophysiological models of FHM, which converge on a cortical hyperexcitability and an increased concentration of glutamate as the common denominator (28, 30, 39). In acute cortical brain slices, we could show an increased and early shift in [K^+^]_e_ in heterozygous mice during CSD. This key finding likely represents the pathophysiological link between increased Na^+^-channel and interneuron activity and an increased susceptibility to CSD, given the important role of K^+^ in initiation and propagation of CSD. Our results are nicely complemented by an accompanying study by Chever et al. (40), demonstrating that CSD can be induced by both optogenetic stimulation of all cortical GABAergic interneurons, exclusively parvalbumin-positive interneurons, and by using Hm1a, a compound specifically enhancing persistent currents carried by Na_V_1.1 channels, thus mimicking the effect of the L1649Q mutation that we observed. Most importantly, they found that optogenetically elicited spiking of interneurons induced a rise of [K^+^]_e_ which was able to trigger CSD. Furthermore, the computational model of the study by Chever et al. (40) confirms that even small changes of persistent currents and a resulting increase in firing frequency, as seen between genotypes at low firing rates in our data, are sufficient to elicit CSD.

In addition to increased interneuron firing per se, a further mechanism leading to K^+^ accumulation is provided by the increased ion fluxes caused by persistent Na^+^ currents, resulting directly in enhanced ion translocation during a single action potential (35, 41). In fact, Na^+^ and K^+^ currents in fast-spiking GABAergic interneurons are exquisitely tuned to minimize kinetic overlap and energy expenditure (42).

### Characteristics of the knock-in FHM3 mouse model.

Our in vivo studies demonstrating an increased susceptibility to CSD are in line with similar observations in other FHM mouse models, including a recently published knock-in mouse model for another FHM3 mutation (p.Leu236Val) (24–26, 31). In contrast to that FHM3 *Scn1a*^L236V^ mouse model, our heterozygous *Scn1a*^L1649Q^ littermates are fully viable and show a normal survival, thus better resembling the human situation in patients with FHM3. We chose to generate a model for the L1649Q mutation, since it is associated with a pure FHM phenotype in humans, whereas the L263V mutation also causes epileptic seizures as part of the clinical spectrum. In line with this pure FHM phenotype, a comprehensive, standardized screening did not reveal substantial phenotypic abnormalities of heterozygous animals. While the sudden death of homozygous *Scn1a*^L1649Q^ knock-in animals could suggest cardiac events, a detailed cardiac characterization found only a mild cardiac phenotype in older heterozygous animals, which was not confirmed in homozygous animals up to P16. The premature death of homozygous animals is in line with the reduced survival of other *Scn1a* knock-in mice, showing a similar peak of mortality (15–17, 43). However, further investigations are needed to clarify the cause of early death of homozygous *Scn1a*^L1649Q^ knock-in mice.

Compared with previous studies on FHM mouse models, we noted several potentially interesting differences. First, in our animals we observed no difference in CSD propagation velocity in vivo, as found for FHM1 and FHM2 (24, 26). This is in line with the recent in vivo characterization of a different FHM3 model (31) and may thus indicate that the mutation predominantly affects CSD initiation. This is supported by the shortened latency of the first recorded CSD in vivo. For the calculation of CSD velocity in our in vitro experiments, we used a shorter distance and included the induction site. Thus, the slight increase in CSD propagation velocity in vitro fits nicely to the in vivo findings. Second, there was no effect of sex. So far, studies on the effect of sex in FHM mouse models are inconsistent. Female FHM1 mice showed an increased CSD frequency and propagation velocity (44). In FHM2, no sex-related differences were reported for the p.Trp887Arg mutation (26), but aged female mice harboring the p.Gly301Arg mutation showed a decreased CSD frequency (27). While a higher prevalence of common migraine in females is well-established (2, 45), our results are in line with previous studies of patients with FHM, demonstrating similar clinical characteristics in female and male patients (46, 47). Third, in our set-up, there was no altered CSD susceptibility in aged animals (9 months). So far, only one other study explored the effect of age on CSD susceptibility, and it found elevated CSD frequency in 14- to 20-month-old male mice (27). Our findings seem to better reflect the human situation, with a decreasing attack frequency in older individuals (46). However, more challenging experimental conditions in older mice, requiring, for example, higher doses of anesthetics, might also have contributed to our observation.

### Premature death of homozygous Scn1aL1649Q knock-in mice and effects of the late Na^+^-current blocker GS967.

The death of homozygous *Scn1a*^L1649Q^ knock-in mice between P16 and P21 is in temporal conjunction with the upregulation of Na_V_1.1 expression in the second and third postnatal week (15, 16, 48). Together with the normal development and growth up to that point, this indicates a causative link between Na_V_1.1 upregulation and the premature death. As a proof-of-concept therapeutic intervention, we targeted the identified Na_V_1.1 gain-of-function using the late Na^+^-current blocker GS967, which normalized sodium channel as well as interneuron function and substantially expanded the lifespan of homozygous *Scn1a*^L1649Q^ knock-in mice.

Since GS967 is not specific to Na_V_1.1 channels and rather specifically blocks persistent but less peak Na^+^ currents (35), we cannot exclude that therapeutic drug effects are also mediated via other Na^+^ channels, such as Na_V_1.6, contributing largely to persistent Na^+^ currents (49).

### Na^+^-channel dysfunction in the context of previous studies.

Our results demonstrate a gain-of-function of Na^+^ channels in *Scn1a*^L1649Q^ knock-in mice as a basis for the increased interneuron firing and inhibitory synaptic activity. The previously reported complete loss of Na_V_1.1 channel function by the L1649Q mutation due to a markedly reduced cell surface expression in mammalian cells (29) could not be confirmed by more recent studies demonstrating robust expression and an overall gain-of-function (30, 35), as in our model. Our data also did not reveal a substantial decrease in peak current amplitudes in heterozygous animals. A depolarizing shift of the inactivation curve, as described for this mutation in tsA201 cells (30), was absent in nucleated patch recordings and was present in only a small amount in Purkinje neurons of homozygous animals. A crucial difference from previous work is that we assessed properties of Na^+^ channels in their natural environment (i.e., a neuron), which might explain the differences from previous work (30, 35).

The Na_V_1.1 gain-of-function might be counterbalanced by the reduced protein expression as observed in 2-month-old mice. In contrast, mRNA levels of heterozygous and WT mice are similar, indicating that the differences in protein levels are either due to reduced translation efficiency or decreased stability of the mutant protein.

### Migraine and epilepsy: 2 diseases, 1 gene.

It is an interesting observation that mutations in the same gene *SCN1A* cause 2 different diseases, with most mutations causing either migraine or epilepsy (50). There are only few exceptions, such as the L236V mutation, which leads to FHM with rare epileptic seizures (51). Previous work, including a knock-in mouse model for a mild epilepsy carrying a neighboring *SCN1A* mutation to L1649Q (R1648H), revealed opposite effects to those observed here, i.e. a loss-of-function of Na^+^ currents and a decreased firing rate of inhibitory interneurons (15–17). We thus conclude that migraine and epilepsy (at least most of them) are caused by opposing effects. Only a strong gain-of-function may be actually able to cause severe epilepsy (52).

### Conclusion.

Our results demonstrate a mechanism for CSD and migraine, as exemplified in a mouse model of FHM3. The model shows excellent overlap with the human phenotype, as illustrated by increased CSD susceptibility and otherwise normal behavior. Using a multimodal electrophysiological approach combining in vivo recordings with analyses in slices and isolated neurons, we provide evidence for an increased activity of fast-spiking inhibitory interneurons and a subsequent increase in extracellular potassium. We believe this represents a novel mechanistic cascade underlying CSD susceptibility. Since CSD is also an important pathophysiological mechanism of other frequent disorders such as ischemic stroke, cerebral hemorrhage, and traumatic brain injury, our findings may be relevant beyond the migraine field.

## Methods

### Generation of Scn1aL1649Q knock-in mice

The *Scn1a*^L1649Q^ knock-in mouse line was generated by introducing the point mutation p.L1649Q/c.4946T>A into exon 27 of *Scn1a*. The targeting strategy, embryonic stem cell targeting, positive and negative selection, and breeding are described in detail in the Supplemental Material. Homozygous (mut/mut) and heterozygous (mut/wt) *Scn1a*^L1649Q^ knock-in animals were used for experiments. WT (wt/wt) littermates were used as controls.

### Phenotypic screening

For survival analysis, litters were monitored between P8 to P21. The pups were weighed daily. Heterozygous *Scn1a*^L1649Q^ knock-in mice (15 males, 15 females) and WT control littermates (12 males, 15 females) underwent a systematic, comprehensive phenotyping screen by the German Mouse Clinic at the Helmholtz Zentrum München (http://www.mouseclinic.de) as previously described (53–56). This screen started at the age of 10 weeks and covered multiple parameters in the areas of behavior, cardiovascular function, clinical chemistry, dysmorphology, energy metabolism, eye analysis and vision, hematology, immunology, neurology, allergy, and pathology. In addition, a cardiovascular screening was performed on homozygous *Scn1a*^L1649Q^ knock-in mice (10 animals) and WT littermates (9 animals) between P10 and P16, including daily electrocardiogram and echocardiography as well as macro- and microscopic work-ups of the heart.

Expression and protein levels of the *Scn1a* gene were assessed in brain tissue from heterozygous and homozygous *Scn1a*^L1649Q^ knock-in animals as well as WT controls (16 days and 2 months) using reverse transcription quantitative PCR and Western blot analysis (see Supplemental Methods).

### Analysis of acutely dissociated neurons

P18 to P25 mice were anesthetized with isoflurane and sacrificed by decapitation according to the 8 2010/63/EU directive on the protection of animals used for scientific purposes. Brain dissection and preparation of the acute culture of cerebellar Purkinje cells were performed as described in the Supplemental Methods and in Carter & Bean (57). Patch clamp recordings of these cells were performed using standard solutions as detailed in the Supplemental Methods.

Data were analyzed with Ana (http://users.ge.ibf.cnr.it/pusch/sframes/Ana.html) and Sigma Plot (SPSS). Various voltage protocols were applied to measure steady-state and kinetic voltage–dependent parameters and were analyzed as detailed in the Supplemental Methods.

### Analysis of acute brain slices

#### Experimental animals.

To label inhibitory neurons and to be able to identify cortical fast-spiking inhibitory neurons, we crossed the *Scn1a*^L1649Q^ knock-in strain with glutamate decarboxylase–GFP (GAD67-GFP) knock-in animals (same C57BL/6 background). In GAD67-GFP knock-in animals, GABAergic inhibitory neurons positive for parvalbumin, calretinin, and somatostatin are colocalized, with parvalbumin-positive cells being the most abundant in the neocortex (40.1% among all GFP positive cells; ref. 58).

#### Preparation and maintenance of slices.

Thalamocortical slices from male and female mice (P14–P20) were obtained with a Microm HM 650V vibratome (Thermo Fisher Scientific) using established procedures (ref. 17, ref. 59, and Supplemental Methods). Horizontal hippocampal slices of mice at the same age were used for recordings in the CA1 (*Cornu Ammonis*) region.

#### Electrophysiological recordings.

Whole-cell patch clamp recordings of inhibitory and/or excitatory neurons within cortex (layers 4 and 5, respectively) and hippocampus were performed at 34°C using a Multiclamp 700B amplifier, a DigiData 1420, and pClamp 10.6 software (all from Molecular Devices) (ref. 17 and Supplemental Methods). For current clamp experiments, cells were held at –70 mV. Cortical inhibitory neurons were identified by their green fluorescence. We only used fast-spiking neurons of layer 4 with homogenous electrophysiological characteristics (AP width, <0.6 ms; APs are followed by a large afterhyperpolarization). For hippocampal fast-spiking neurons, we used only fast-spiking neurons with typical electrophysiological characteristics as mentioned above and typical morphological properties (located at the border of the *stratum oriens* and the *stratum pyramidale*, see also Supplemental Figure 7).

To record sIPSCs and mIPSCs, the AMPA (α-amino-3-hydroxy-5-methyl-4-isoxazolepropionic acid) and Kainate receptor antagonist 6,7-dinitroquinoxaline-2,3-dione (NBQX; 10 μM, Sigma-Aldrich) and the NMDA-receptor antagonist (2R) amino-5-phosphonovaleric acid (APV, 30 μM) were added to the standard aCSF as described in detail in the Supplemental Methods. The membrane voltage was clamped to –70 mV and IPSCs were recorded over 5-minute epochs. For recording ramp Na^+^ currents, voltage ramps from –80 to 20 mV with a velocity of 25 mV/s were used. aCSF and internal solutions supplemented with K^+^ and Ca^2+^ blockers were used as described in the Supplemental Methods.

Nucleated patch recordings of hippocampal fast-spiking basket cells in the hippocampal CA1 (at the border between *stratum oriens* and *stratum pyramidale*) of acute slices were performed using a multiclamp 700B amplifier, a Digidata 1420, and pClamp 10 data acquisition software (all from Molecular Devices) as described before (17). Traces were displayed off-line with Clampfit software of pClamp 10.0 (Molecular Devices).

The voltage clamp protocols and data analysis for transient Na^+^ currents recorded from neurons are described in the Supplemental Methods.

#### Cortical spreading depression.

Acute coronal slices were perfused with aCSF containing a modified concentration of 3.5 mM KCl as described in the Supplemental Methods. A quantity of 200 mM KCl supplemented with 0.1% Fast Green was applied with a glass pipette (0.2–0.5 MΩ) onto the slice surface on layer 2/3 using a PDES-02DX pneumatic drug ejection system (Npi Electronic) to eject pulses (0.5 bar) with a duration of 400 ms to elicit a CSD as previously described (60). Electrodes were at least 1000 μm apart from the puffing electrode. The K^+^ selective and the extracellular local field potential pipettes were placed at a distance less than 100 μm from each other. CSD generation was visualized using a CCD Camera (Retiga Electro). Electrophysiological signals were recorded using an EPC 10 USB Quadro System (HEKA) and Patchmaster next software (HEKA). DC–extracellular potential recordings were performed using borosilicate glass micropipettes (~0.5 MΩ) filled with aCSF. K^+^ selective electrodes were built out of borosilicate glass capillaries as previously described (61) and as detailed in the Supplemental Methods (see also Supplemental Figure 5A).

### In vivo cortical spreading depression

Experiments were performed in both male and female 2-month-old and 9-month-old heterozygous *Scn1a*^L1649Q^ knock-in mice. WT littermates at the same age were used as controls. Mice were anesthetized, placed in a stereotactic frame (Kopf Instruments), and monitored for physiological conditions (for details see Supplemental Methods and Supplemental Table 7).

Three holes were drilled in the skull over the left hemisphere at the following coordinates from bregma: lateral 1 mm, ap 1.7 mm (position 1); lateral 1.5 mm, ap -1 mm (position 2); lateral 3.5 mm, ap –3.5 mm (position 3) (see Supplemental Figure 8).

An Ag/AgCl electrode was placed subcutaneously at the neck as a reference electrode. To record CSD, glass pipettes (borosilicate glass) were fixed on a micromanipulator, filled with saline, and placed at position 1 and position 2, 200 μm below the dura. After electrode insertion, DC potential and electrocorticogram were recorded for 5 minutes as baseline prior to cortical stimulation.

To determine CSD frequency, a cotton ball (diameter 3 mm) soaked with 300 mM KCl was placed at position 3 on the cortex for 1 hour. CSD threshold was determined by electrical stimulation with a concentric Tungsten electrode (MicroProbes) placed at position 3. A stimulus isolator unit (Digitimer) was used to generate pulses of increasing intensity (1–100 μC). Pulses were applied with an interval of 3 minutes until a CSD was evoked. The minimal charge intensity needed to evoke a CSD was taken as CSD threshold. As positive control, a CSD was elicited with 3 M KCl at the end of each experiment. Signals were amplified and low pass filtered (Warner Instruments), continuously digitized, and recorded using PowerLab and LabChart (AD Instruments). To obtain CSD propagation velocity, the distance between the recording electrodes was measured and divided by the latency of a CSD between the electrodes.

### Administration of GS967

We studied the effect of the late Na^+^-current blocker GS967 (MedChemExpress) on hippocampal inhibitory neurons, on CSD initiation, and on the survival of homozygous *Scn1a*^L1649Q^ knock-in mice. For eliciting Na^+^ ramp currents without and with 3 μM GS967, voltage ramps from –80 to 20 mV with a velocity of 25 mV/s were used as described in detail in the Supplemental Methods. To record the number of action potentials with and without 3 μM GS967, current injections up to 0.95 nA were used. To test the effect on CSD initiation we used a concentration of 5 μM GS967.

GS967 treatment of homozygous animals commenced at P14. The compound was mixed with the chow (8 mg GS967/kg) and added to the drinking water (2 μM) as previously described (37). Control mice received regular chow and water. Until complete weaning, pups ingested the drug also via the milk of the mother. Litters were randomized to GS967 treatment or control group after genotyping and ascertaining the presence of homozygous animals.

### Statistics

For all experiments, the investigator was blinded to the genotype. Statistics were done using Prism 7.04 (GraphPad Software), SigmaStat 3.1 (Statcon), and R (version 3.2.3; ref. 62). The following tests were used as indicated in the respective figure legends or in the text: Mann-Whitney rank-sum test for unpaired data sets of wt/wt and mut/wt animals, ANOVA on ranks (Kruskal-Wallis test) with Dunn’s post hoc test for comparing more than 2 groups, 2-tailed *t* test or 1-way ANOVA when 2 data sets of unpaired groups were normally distributed. Survival was analyzed using Kaplan Meier curves and log rank test for group comparison. For testing the effect of GS967 on the Na^+^ ramp currents and the area under the curve of input-output curves (Figure 8D), 2-way ANOVA was used. A *P* value less than 0.05 was considered significant.

In the phenotypic screen, tests for genotype effects were done using 2-tailed *t* test, Wilcoxon rank sum test, linear models, ANOVA, and post-hoc tests, or Fisher’s exact test depending on the type and assumed distribution of the parameter. There was no correction for multiple testing (about 500 parameters belonging to 14 different disease areas are measured during the primary screening) performed in this exploratory analysis. A statistically significant difference might be a hint for a new phenotype, but is not per se physiologically relevant, and the possibility for false-positive results remain.

Box-and-whisker plots show medians (lines), lower and upper quartiles, and minimums and maximums. For all statistical tests, significance with respect to control is indicated on the figures using the following symbols: *P < 0.05, **P < 0.01, ***P < 0.001.

### Study approval

Experiments were approved by the local animal care and use committees (Regierungspraesidium Tuebingen, Germany; Regierungspraesidium Oberbayern, Germany; Italian Ministry of Health and Local Organism in charge of animal welfare).

## Author contributions

EA, UBSH, RB, BG, TVW, TF, NP, MP, PG, and HL designed the experiments. EA, UBSH, RB, DM, BG, NV, PL, IZ, SB, and PG performed the experiments. EA, UBSH, RB, DM, BG, TVW, TF, NP, MP, PG, and HL analyzed data. NS, HF, VGD, and MHA designed, performed, and analyzed the phenotypic screening. MD revised the manuscript. EA and UBSH wrote the manuscript and all authors revised the manuscript. Order of co–first authors was determined by actual contribution to the paper.

## Supplementary Material

Supplemental data

## Version 1 09/21/2021

In-Press Preview

## Version 2 11/01/2021

Electronic publication
