# Supplementary material for: Hyperexcitable interneurons trigger cortical spreading depression in an Scn1a migraine model
Source: J Clin Invest. 2021 Nov 1;131(21):e142202. doi: 10.1172/JCI142202 (PMC8553559; doi:10.1172/JCI142202)
Supplement: Supplemental data [file jci-131-142202-s202.pdf]

SUPPLEMENTARY METHODS

Generation of *Scn1a*^{L1649Q} knock-in mice

Mouse genomic DNA was derived from the C57Bl/6J RPCIB-731 BAC library. The final vector carried a puromycin resistance gene as positive selection marker flanked by F3-sites and a thymidine kinase for negative selection.

The vector was linearized and transfected via electroporation into C57Bl6/N E14 embryonic stem cells (ES). After positive and negative selection (puromycin/ganciclovir) ES were tested for homologous recombination by southern blot analysis. Genomic DNA from ES was digested with *AvrII* and *MscI.* Hybridisation with external probes gave 25.6 kb and 15.4 kb fragments (*AvrII*) and 7.4 kb / 9.3 kb fragments (*MscI*) for wildtype and targeted allele, respectively (overview in Figure 1B).

The presence of the L1649Q point mutation was verified by sequencing analysis. Correctly targeted ES cells were injected in BALB/c blastocysts. The blastocysts were transferred into pseudopregnant NMRI-females. Highly chimeric G0 males were bred with C57BL/6N females carrying a FLP recombinase to delete the Puromycin cassette. *Scn1a*^{L1649Q} knock-in mice in which the Puromycin cassette was successfully deleted were backcrossed with C57BL/6N for more than six generations. Genotyping was done by PCR using the following primers: 5'-TAGTGTGCAAGCTTGAGAACG-3' and 5'-TCAGCTCCTTCAGTCCTTGC-3'. The generation was done by TaconicArtemis.

Experimental animals were housed in groups of max. 5 animals in individually ventilated cages (Techniplast) with a 12 hours light/dark cycle. Animals had ad libitum access to food and water. The room temperature was 21°C with a relative humidity of 45-65%.

Reverse transcription quantitative PCR (RT-qPCR)

For qPCR analysis brain tissue from heterozygous and homozygous *Scn1a*^{L1649Q} knock-in animals as well as wildtype controls (16 days or 2 months old) was used. The hemispheres were separated, and cortices and brainstems were isolated. Dissected tissue samples were homogenized in 500 µl Trizol (Qiagen), after homogenization samples were filled up to 1 ml with Trizol.

Total RNA isolation was performed using the RNeasy Lipid Tissue Mini Kit (Qiagen). RNA concentrations were measured using a Nanodrop ND-1000 Spectrophotometer (Peqlab). 1 µg of RNA was reverse transcribed using the Omniscript RT Kit (Qiagen) according to the manufacturer's instructions using oligo-dT-primer. Quantitative polymerase chain reaction was performed with a LightCycler 480 II (Roche) using Brilliant II SYBR Green qPCR Master Mix (Agilent). All samples were analysed in triplicates and for each primer negative controls (-RT and H_2O) were performed. The following primer sequences were used for *Scn1a*: 5'-GAA

GGGAATAGACTGACATATGAAAA-3' (forward) and 5'-GGGAACCACGAATGCTTAAC-3' (reverse) and GADPH: 5'-ATTGTCAGCAATGCATCCTG-3' (forward) and 5'-ATGGACTGT GGTCATGAGCC-3' (reverse).

For quantification of changes in gene expression we first calculated dCt values using GAPDH as housekeeping gene and then used the 2^{-ddCt} – method (1) to calculate fold changes normalized to wild type expression.

Western blot analysis

Brain tissue of *Scn1a*^{L1649Q} knock-in mice (16 days or 2 months old) was used for western blot analysis. The hemispheres were separated, cortices and brainstem were isolated. Dissected tissue samples were homogenized in RIPA buffer supplemented with protease and phosphatase inhibitor cocktail (Roche). Cell debris in the homogenate was removed by a short spin (1200 g, 10 min, 4°C) and the supernatant was centrifuged again (25000 g, 30 min, 4°C). The pellet was resuspended in 500 µL RIPA buffer to obtain a membrane enriched fraction. 15 µg protein of membrane enriched fraction was separated on a NuPAGE tris-acetate 3-8 % gel and transferred to a nitrocellulose membrane. Membranes were blocked overnight in TBS-T with 4 % non-fat milk powder and incubated with the primary antibody at 4° C over night, followed by the appropriate horseradish peroxidase (HRP)-conjugated secondary antibody, incubated at room temperature for 1 hour. As primary antibodies we used rabbit anti- $Na_V1.1$ antibody (1:500, Alomone, Cat# ASC-001), which has been validated in a $Na_V1.1$ knock out model (2), and rabbit anti-actin-antibody (1:1000, Sigma, Cat# A2066) as loading control. Bands were detected using the Immobilon Western ECL detection reagent (Millipore) with a Fusion Fx7 imager. For quantification, the density of the protein bands was measured using the gel analysis tool in ImageJ. The signal intensity of each protein band was normalized to the respective beta-actin band to adjust for any minor differences in total protein loading.

Analysis of acutely dissociated neurons

Dissection. P18-P25 mice were anaesthetized with isoflurane and sacrificed by decapitation according to the directive 8 2010/63/EU on the protection of animals used for scientific purposes. Mouse brain dissection and preparation of the acute culture of cerebellar Purkinje cells were performed by the procedure described below and as described previously (3). The brain was quickly removed and placed into ice-cold solution (110 mM NaCl, 2.5 mM KCl, 10 mM Hepes, 25 mM glucose, 75 mM sucrose, 7.5 mM $MgCl_2$, 30 mM Mannitol; pH 7.4 with NaOH; osmolarity ~ 357 mOsm). Meninges and vessels were peeled off and the cerebellum was isolated and cut with a scalpel into ~400 µm slices. The tissue was treated with protease solution (82 mM Na_2SO4, 30 mM K_2SO_4, 5 mM $MgCl_2$, 10 mM Glucose, 10 mM Hepes, 3 mg/mL Protease XXIII Sigma; pH 7.4 with NaOH; osmolarity ~366 mOsm) for 20 min at room

temperature which was then replaced by ice-cold Inhibition solution (82 mM Na_2SO_4, 30 mM K_2SO_4, 5 mM $MgCl_2$, 10 mM Glucose, 10 mM Hepes, 1 mg/mL trypsin inhibitor, 1 mg/ml BSA; pH 7.4 with NaOH; osmolarity ~ 366 mOsm). Cerebellar chunks were kept on ice in this solution until immediately before use. To release individual cells, the tissue was passed through fire-polished Pasteur pipettes with degrading tip size (modified from 3). Purkinje cells could be recognized by their large size and a single large dendritic stump (Figure 3A).

Patch clamp recordings. The standard extracellular recording solution contained (in mM): 20 NaCl, 55 CsCl, 20 TEACl, 1 $CaCl_2$, 2 $MgCl_2$, 1 $CdCl_2$, 116 glucose, 10 Hepes, pH 7.35 with NaOH. The intracellular solution contained: 50 CsCl, 80 CsF, 11 EGTA, 1 $CaCl_2$, 10 Hepes, pH 7.3 with Tris. All salts were purchased from Sigma. Currents were recorded with an Axon 200 amplifier at 21 +/- 1 °C (Peltier controlled) using the custom GePulse acquisition software (http://users.ge.ibf.cnr.it/pusch/sframes/Gepulse.html). Borosilicate pipettes were pulled to a resistance of 1.5-2.5 MOhm. Recordings with series resistance errors >5 mV at peak current were discarded.

Data analysis. Data were analyzed with the program Ana (available at http://users.ge.ibf.cnr.it/pusch/sframes/Ana.html) and Sigma Plot. Various voltage protocols were applied to measure steady state and kinetic voltage dependent parameters; holding potential was -90 mV throughout. Leak and capacitive currents were subtracted using a P/4 protocol. The standard IV-protocol consisted of 15 ms long pulses to voltages ranging from -70 mV to +5 mV in 5 mV steps. Steady-state activation was determined by fitting the peak current-voltage (I-V) relationship with the equation:

$$I(V) = \frac{g_{max}(V - V_{rev})}{1 + exp^{-z_{act}(V-V_{1/2})F/RT}}$$

with V_{rev} being the reversal potential, g_{max} the maximum conductance, $V_{1/2}$ the potential for half-maximal activation, z_{act} the apparent gating valence, R the gas constant and T the temperature. The time course of inactivation was analyzed by fitting the decaying phase of the sodium current with a double exponential function of the form:

$$I(t) = I_\infty + I_f e^{-\frac{t}{\tau_f}} + I_s e^{-\frac{t}{\tau_s}}$$

where I_f and τ_f are respectively the fast component of inactivation and the fast time constant, while I_s and τ_s are respectively the slow component and the slow time constant. These values were further combined to determine the relative area of the slow component of inactivation:

$$A_{rel} = \frac{I_s \tau_s}{I_s \tau_s + I_f \tau_f}$$

Steady-state inactivation was measured applying 100 ms conditioning pulses to various voltages followed by a test pulse to –10 mV, and currents were fitted with the equation:

$$I(V) = \frac{I_{max}}{1 + exp^{(V - V_{1/2})/k}}$$

where V represents the pre-pulse potential, I_{max} is the maximal current, $V_{1/2}$ the voltage of half-maximal inactivation, and k the slope factor.

The time course of recovery from inactivation at -120 mV was measured by repolarizing the cell to -120 mV for a variable time after a 100 ms pulse to 0 mV, and assessing channel availability by a final test-pulse to -10 mV. Peak-currents at the final test pulse were fitted to a double exponential function:

$$I(t) = I_{max} - I_f e^{-\frac{t}{\tau_f}} - I_s e^{-\frac{t}{\tau_s}}$$

where I_f and τ_f are the fast component of recovery from inactivation and the fast time constant, respectively, while I_s and τ_s are the slow component and the slow time constant, respectively. Steady-state currents (see Figure 3F) were calculated at the end of the 15 ms test pulse to -25 mV.

Analysis of acute brain slices

Preparation and maintenance of slices. Thalamocortical slices from male and female mice at postnatal day 14-20 were obtained with a Microm HM 650V vibratome (Thermo Fisher Scientific Inc.) using procedures as described before (4, 5). Horizontal hippocampal slices of the same age were used for recordings in the CA1 region. Animals were anesthetized with Isofluorane (CP-Pharma) and promptly decapitated. Brains were removed quickly and cut in ice-cold artificial cerebrospinal fluid (aCSF) with the following composition (in mM): 125 NaCl, 25 $NaHCO_3$, 2.5 KCl, 7 $MgCl_2$, 2 $CaCl_2$, 1.25 NaH_2PO_4, 10 Glucose (pH 7.4, equilibrated with 95% O_2/5% CO_2). Slices were stored to recover at 36°C for 1 h before recordings. For storage and recording the following solution was used (in mM): 125 NaCl, 25 $NaHCO_3$, 2.5 KCl, 1 $MgCl_2$, 2 $CaCl_2$, 1.25 NaH_2PO_4, 10 Glucose (pH 7.4, equilibrated with 95% O_2/5% CO_2) (standard aCSF).

Immunohistochemistry. For morphological studies patched cells were filled with 0.2% biocytin (Supplementary Figure 8). Slices were fixed for one hour with 4% paraformaldehyde at room temperature. After washing steps with PBS slices were incubated for one hour at room temperature with cy3-conjugated Streptavidin (Jackson ImmunoResearch Laboratories, Inc.; dilution 1:600). Slices were stained with DAPI (Sigma-Aldrich, St.Louis, MO, USA; dilution 1:10000) to identify the nuclei. After washing slices were air-dried and mounted with Fluoromount (Sigma-Aldrich) and visualized on an Axiophot 2 (Zeiss).

Electrophysiological recordings. Whole-cell patch clamp recordings of inhibitory and/or excitatory neurons within cortical layer 4 and 5 or hippocampus were performed at 34°C using a Multiclamp 700B (Molecular devices) amplifier, a DigiData 1420 (Molecular devices) and

pClamp 10.6 software (Molecular devices) as described before (5). Slices were positioned in a submerged-type recording chamber (Scientifica, United Kingdom), continuously superfused with aCSF and visualized with a BX61WI Microscope (Olympus). Pipettes were pulled from borosilicate glass (Science products) using a Sutter P97 Puller (Sutter Instruments), with resistances of 3-5 MΩ. For current clamp experiments cells were held at -70 mV, the patch pipette solution contained (in mM): 5 KCl, 4 ATP-Mg, 10 phosphocreatine, 0.3 GTP-Na, 10 HEPES, 125 K-Gluconate, 2 $MgCl_2$, 10 EGTA and 0.2% Biocytin with a final pH of 7.2 and an osmolarity of 290 mOsm/kg.

To record spontaneous and miniature inhibitory postsynaptic currents (IPSCs), the AMPA and Kainate receptor antagonist 6,7-dinitroquinoxaline-2,3-dione (NBQX; 10 µM, Sigma-Aldrich) and the NMDA-Receptor antagonist (2R)-amino-5-phosphonovaleric acid (APV, 30 µM) were added to the standard aCSF; patch pipettes contained (in mM): 105 CsCl, 35 CsOH, 10 HEPES, 10 EGTA, 10 phosphocreatine, 4 ATP-Mg, 0.3 GTP-Na, 14 D-Mannitol, 0.2% Biocytin. Membrane voltage was clamped to -70 mV and IPSCs were recorded over 5 min epochs. Current recordings were sampled at 100 kHz and the data were filtered at 30 kHz. Series resistance (<20 MΩ) was monitored during the experiment. Cells showing unstable series resistance or resting membrane potential were discarded. For analysis of events, traces were filtered at 3 kHz using software Bessel filter (Clampfit, Molecular devices). mIPSCs and sIPSCs were identified using event detection in Clampfit. Each automatically identified event was manually confirmed.

Nucleated patch recordings of fast spiking basket cells of the hippocampal CA1 region in acute slices were performed using a multiclamp 700B amplifier, a Digidata 1420 and pClamp 10 data acquisition software (Molecular devices) as has been described before (5, 6). Leakage and capacitive currents were automatically subtracted using a pre-pulse protocol (-P/4). Currents were filtered at 5 kHz and digitized at 20 kHz. All recordings were performed at room temperature of 21–23°C. Borosilicate glass pipettes had a final tip resistance of 1-2 MΩ when filled with internal recording solution. The aCSF for recording Na^+-currents in nucleated patches in acute slices contained (in mM): 125 NaCl, 25 $NaHCO_3$, 2.5 KCl, 1.25 NaH_2PO_4, 2 $CaCl_2$, 1 $MgCl_2$, 25 glucose, bubbled with 95% O_2 and 5% CO_2. TEA (20 mM) was added to block voltage-gated K^+ channels. Pipettes were filled with Cs^+ rich internal solution, containing (in mM): 140 CsCl, 10 EGTA, 2 $MgCl_2$, 2 ATP-Na_2, 10 HEPES, pH adjusted to 7.3 with CsOH. For recording the ramp sodium current in hippocampal inhibitory neurons (fast spiking basket cells located at the border between *Stratum pyramidale* and *Stratum oriens*) in acute slices, the aCSF contained the following (in mM): 50 NaCl, 90 TEA-Cl, 10 HEPES-free acid, 2 $CaCl_2$, 2 $MgCl_2$, 3.5 KCl, 3 CsCl, 0.2 $CdCl_2$, 4 4-AP, and 25 glucose. pH was adjusted to 7.4 using NaOH. The intracellular solution contained the following (in mM): 110 CsF, 10 HEPES-Na, 11 EGTA, 2 $MgCl_2$, 0.5 GTP-Na, and 2 ATP-Na_2. pH was adjusted to 7.3 with CsOH. For eliciting

ramp sodium currents, voltage ramps from -80 to 20 mV with a velocity of 25 mV/s were used. In each case, currents were recorded in aCSF and in the presence of TTX to subtract remaining potassium or calcium currents. The integral of the ramp currents was calculated using Origin 9.1 software (OriginLab Cooperation, Northhampton, USA).

Voltage clamp protocols and data analysis for transient Na^+-currents recorded from neurons. For the used voltage clamp protocols, recorded currents from nucleated patches were averaged up to 10 times to obtain homogeneous results. The activation curve (conductance–voltage relationship) was derived from the current–voltage relationship that was obtained by measuring the peak current at various step depolarizations (7.5 mV steps) from the holding potential of -90 mV. Current-Voltage (I-V) curves were obtained by plotting the maximal current of every trace *vs* the voltage. After normalization to the maximal current amplitude the following Boltzmann function was fit to the data points:

$$I(V) = \frac{g \cdot (V - V_{rev})}{\{1 + \exp[(V - V_{1/2}) / k_V]\}}$$

with I being the recorded current amplitude at test potential V, V_{rev} the Na^+ reversal potential, g the unitary conductance, $V_{1/2}$ the voltage of half-maximal activation and k_V a slope factor. The conductance was calculated directly by using the equation with the obtained reversal potentials:

$$g(V) = \frac{I}{(V - V_{rev})}$$

with g being the conductance, I the recorded current amplitude at test potential V, and V_{rev} the obtained Na^+ reversal potential.

The following Boltzmann function was fit to the obtained data points:

$$g(V) = \frac{g_{\max}}{\{1 + \exp[(V - V_{1/2}) / k_V]\}}$$

with g being the conductance, I the recorded current amplitude at test potential V, V_{rev} the Na^+ reversal potential, g_{max} the maximal conductance, $V_{1/2}$ the voltage of half-maximal activation and k_V a slope factor. Steady-state inactivation was determined using 300 ms conditioning pulses to various potentials followed by the test pulse to -20 mV at which the peak current reflected the percentage of non-inactivated channels. A standard Boltzmann function was fit to the inactivation curves:

$$I(V) = \frac{I_{\max}}{\{1 + \exp[(V - V_{1/2}) / k_{\mathrm{V}}]\}}$$

with I being the recorded current amplitude at the conditioning potential V, I_{max} being the maximal current amplitude, $V_{1/2}$ the voltage of half-maximal inactivation and k_V a slope factor.

The membrane was depolarized to various test potentials from a holding potential of -90 mV to record Na^+-currents. A second-order exponential function was best fit to the time course of fast inactivation during the first 70 ms after onset of the depolarization, yielding two time constants. The weight of the second slower time constant was relatively small. Only the fast time constant, named τ_h, was used for data presentation in Supplementary Table 6. Recovery from fast inactivation was recorded from cells depolarized to -20 mV for 100 ms to inactivate all Na^+-channels and then repolarized to -90 mV recovery potential for increasing duration. A second-order exponential function with an initial delay was best fit to the time course of recovery from inactivation. The faster time constant with the much larger relative amplitude, τ_{rec}, is shown in Supplementary Table 6.

Traces were displayed off-line with Clampfit software of pClamp 10.0 (Molecular Devices).

Cortical Spreading Depression. Coronal slices used for CSD recordings were stored and perfused in a modified aCSF with the following composition (in mM): 124 NaCl, 26 $NaHCO_3$, 3.5 KCl, 1 $MgCl_2$, 2 $CaCl_2$, 1.2 NaH_2PO_4, 20 Glucose (pH 7.4, equilibrated with 95% O_2/5% CO_2). Each slice was exclusively used for a single CSD induction.

K^+ selective electrodes were built out of borosilicate glass capillaries, which were first cleaned with 1 M HCl overnight and subsequently rinsed in 70% ethanol. After drying them completely at 120°C for 6-8 hours, the pulled pipettes were pre-treated with dimethylchlorosilane vapors (Sigma-Aldrich, USA), dried at 220°C for 30 min. Finally, the pipette was backfilled with NaCl and a small droplet of the K^+ ionophore I-cocktail B (Fluka, Sigma-Aldrich, USA) was absorbed into the broken pipette tip (5-10 µm) and the tip was filled with the ionophore to 1-2 mm. Each electrode was calibrated using solutions of different K^+ concentrations (0.1, 1, 3.5, 10, and 100 mM KCl added to the aCSF), and only electrodes with slopes between 52 and 58 mV per log change in $[K^+]$ were selected for K^+ concentration measurement (see Supplementary Figure 5A). After each recording the electrodes were re-calibrated and checked for maintaining the correct slope. The baseline of the $[K^+]_e$ was determined by averaging the first 10 s after the KCl puff. The beginning of the extracellular K^+ increase was defined as a +0.1 mM change of baseline concentration. The inflection-point of the K^+ signal was estimated using the peak of the 1st derivative of the K^+ signal (see Supplementary Figure 5B).

CSD elicited by puff application of 200 mM KCl were monitored via an intrinsic optical signal (IOS). Each movie capturing the IOS was acquired with an Ocular Image Acquisition Software and had a fixed frame rate of 10 frames per second. The duration of each movie varied from 85.4 to 88.5 s. Exposure time was 100 ms. A custom-made ImageJ Fiji macro was designed for movie processing. The contrast was adjusted manually for each series of images. The very first frame was taken just before CSD induction and used for subtracting image background. A horizontal line was drawn starting at the manually marked site of KCl release. The Plot Profile Function subsequently generated a data sheet containing the values of pixel intensities along

the drawn line and for each frame. The highest intensity values enabled the identification of the CSD wave front. A custom-made Matlab Live Script was used to calculate the propagation velocity. For each slice, speed was quantified using the average of ten different time points. CSD was called aborted when the CSD decelerated and stopped within <900 µm away from the initiation site.

Cortical spreading Depression in vivo

Experiments were performed in both male and female, 2-months and 9-months old heterozygous *Scn1a*^{L1649Q} knock-in mice (bodyweight 25 – 37 g according to age and sex). Wildtype littermates at the same age were used as controls. Mice were anesthetized, placed in a stereotactic frame (Kopf Instruments) and monitored for physiological conditions. Briefly, anesthesia was induced using 4% isoflurane inhalation followed by an intraperitoneal injection of 0.05 mg/kg fentanyl (Janssen-Cilag), 5 mg/kg midazolam (Braun), and 0.5 mg/kg medetomidine (Pfizer); anesthesia was maintained by hourly injections containing one-third of the initial dose of anesthetics. After induction, mice were intubated and mechanically ventilated (Minivent, Hugo Sachs) with a 70%/30% air/oxygen gas mixture and end-tidal pCO_2 was measured using microcapnometry (CI240, Columbus Instruments). A thermostatically regulated feedback-controlled heating pad was used to maintain a body temperature of 37°C. A catheter was put in the femoral artery to measure blood pressure and analyze blood gases. Heart rate and oxygen saturation were measured by a paw sensor (Kent scientific). Regional cerebral blood flow was measured continuously using a flexible laser Doppler flowmetry probe (PeriFlux System 5000, Perimed) glued onto the left temporal bone above the territory of the left middle cerebral artery. All values were within their respective physiological range throughout the experiments. The condition of the cortex was monitored during the whole experiment using a surgical microscope, animals with lesions were excluded.

Supplementary References

1. Livak KJ, and Schmittgen TD. Analysis of relative gene expression data using real-time quantitative PCR and the 2(-Delta Delta C(T)) Method. *Methods.* 2001;25(4):402-8.

2. Kalume F, et al. Sleep impairment and reduced interneuron excitability in a mouse model of Dravet Syndrome. *Neurobiol Dis.* 2015;77:141-54.

3. Carter BC, and Bean BP. Sodium entry during action potentials of mammalian neurons: incomplete inactivation and reduced metabolic efficiency in fast-spiking neurons. *Neuron.* 2009;64(6):898-909.

4. Agmon A, and Connors BW. Thalamocortical responses of mouse somatosensory (barrel) cortex in vitro. *Neuroscience.* 1991;41(2-3):365-79.

5. Hedrich UB, et al. Impaired action potential initiation in GABAergic interneurons causes hyperexcitable networks in an epileptic mouse model carrying a human Na(V)1.1 mutation. *J Neurosci.* 2014;34(45):14874-89.

6. Martina M, and Jonas P. Functional differences in Na+ channel gating between fast-spiking interneurones and principal neurones of rat hippocampus. *J Physiol.* 1997;505 (Pt 3):593-603.

Supplementary Table 1: **Comprehensive phenotypic analysis of heterozygous *Scn1a*^{L1649Q} knock-in mice**

	Age [weeks]	Method/parameter	Phenotype (wt/wt vs mut/wt)
**Behaviour**	9-10	Open field:<br>- Exploration/rearing activity, n<br>- anxiety-related behaviour/time spent in centre, %	 148.3±35.24 vs. 175.2±23.33, p=0.002<br>25.03±12.10 vs. 30.09±9.35, p=0.038
		Acoustic startle & prepulse inhibition	No difference
**Neurology**	9-10, 17	Modified SHIRPA<br>Grip strength<br>Rotarod<br>Auditory brainstem response	No difference
**Clinical Chemistry and haematology**	11, 14,19	Fasting glucose, mmol/l<br>Fasting glycerol, mmol/l<br>Sodium, mmol/l<br>Creatinine, µmol/l	9.27±1.35 vs.10.60±1.59, p=0.001<br>0.274±0.062 vs.0.304±0.039, p=0.034<br>149.5±2 vs. 150.5±2, p=0.045<br>10.09±1.01 vs. 11.23±1.38, p=0.038#
**Nociception**	12	Hot plate	No difference
**Dysmorphology**	12, 17	Anatomical observation	No difference
		Dual-energy X-Ray absorptiometry (DEXA):<br>- body weight, g<br>- bone mineral content, mg<br>- bone mineral density, g/cm^2<br>- lean body mass, g<br>- further DEXA parameters	 <br>24.2±4.8 vs. 23.8±3.5, p = 0.024<br>544.6±56 vs. 529.9±41, p = 0.03<br>73.7±2.9 vs. 72±2.3, p = 0.008<br>20±3.1 vs. 19.5±2.6, p = 0.012<br>No difference
**Allergy**	12, 19	Ig-ELISA	No difference
		Transepidermal water loss	
		Body surface temperature	
**Energy Metabolism**	13, 18	qNMR<br>indirect calorimetry	No difference<br>No difference
**Cardiovascular**	15	Echocardiography	No difference
		Electrocardiogram	**For details see Supplementary Tables 2 and 3**
**Eye**	16	Scheimpflug imaging	No difference
		OCT	No difference
		Laser interference biometry	No difference
		Virtual drum, cyc/deg	0.423 ± 0.014 vs. 0.383 ± 0.025
**Immunology**	19	Flow cytometry of PBCs	No difference
**Pathology**	19	Macroscopy & histology	No difference

Values are expressed as mean ± SD or median [IQR]. # Significant difference only in male animals
OCT (Optical coherence tomography); qNMR (quantitative nuclear magnetic resonance); PBCs (peripheral blood cells).

Supplementary Table 2: Electrocardiography parameters of heterozygous *Scn1a*^{L1649Q} knock-in mice

	Female		Male		Female	Male	overall
	**wt/wt**	**mut/wt**	**wt/wt**	**mut/wt**	p-value	p-value	p-value
	n=15	n=15	n=9	n=15			
**Heart rate, bpm**	791.2 [770.2,797.4]	789.1 [770.7,794.8]	771.2 [755.1,784,3]	772.3 [752.1,787.2]	0.653	0.804	0.607
**RR, ms**	75.8 [75.2, 77.9]	76 [75.5, 77.8]	77.8 [76.5, 79.5]	77.7 [76.2, 79.8]	0.616	0.872	0.589
**PR, ms**	31.3 [30.6, 32.2]	32 [30.4, 32.5]	30.6 [30, 31.3]	30.9 [30, 32.3]	0.532	0.568	0.65
**QRS, ms**	11.7 [11.3, 12.8]	12.3 [11.6, 13.2]	13.2 [12.2, 13.5]	11.8 [11.2, 12.2]	0.429	0.065	0.559
**QT, ms**	18.9 [17.9, 20.4]	20.3 [17.6, 21.8]	20 [19.4, 20.8]	19.3 [17.7, 20.4]	0.506	0.269	0.997
**QTc, ms**	69 [63.9, 73.7]	72.6 [63.2, 77.8]	72.3 [70, 77.3]	70.1 [63.8, 73.8]	0.506	0.371	0.99
**QT dispersion, ms**	1.9 [1.4, 2.7]	0.7 [0.4, 1.1]	0.8 [0.6, 0.9]	1 [0.7,1.4]	**0.003**	0.219	0.039
**QTc dispersion, ms**	6.9 [4.8, 9.8]	2.7 [1.1, 5]	2.7 [1.8, 3.2]	3.7 [2.8, 5.8]	**0.006**	0.178	0.075
**P, ms**	10.6 [9.1, 10.8]	10.1 8.8, 11.8]	11 [10.9, 11.4]	11.1 [9.2, 12.4]	0.83	0.895	0.656

Data obtained in 15 weeks old animals. Values are expressed as Median [1st quartile, 3rd quartile].

Supplementary Table 3: Heart rate variability analysis of heterozygous *Scn1a*^{L1649Q} knock-in mice

	Female		Male		Female	Male
	**wt/wt**	**mut/wt**	**wt/wt**	**mut/wt**	**p-value**	**p-value**
	n=15	n=15	n=9	n=15		
**RR interval, ms**	76.283 ± 1.655	76.071 ± 1.753	77.167 ± 2.291	77.357 ± 1.717	0.931	0.899
**SDRR, µs**	682.847 ± 318.567	917.014 ± 412.846	534.289 ± 230.388	818.771 ± 415.977	0.149	0.068
**RMSSD, µs**	782.187 ± 398.178	808.6 ± 273.736	658.767 ± 194.928	870.921 ± 206.294	0.337	0.051
**CVRR**	0.009 ± 0.004	0.012 ± 0.006	0.007 ± 0.003	0.011 ± 0.005	0.163	0.078
**SD1, µs**	578.760 ± 317.144	584.693 ± 211.348	473.911 ± 138.493	628.479 ± 147.643	0.471	**0.044**
**SD2, µs**	718.927 ± 434.600	1112.279 ± 637.724	564.567 ± 343.221	937.779 ± 629.315	0.098	0.102

Data obtained in 15 weeks old animals. Values are expressed as mean ± SD.
SDRR (standard deviation of RR interval), RMSSD (root-mean square differences of successive RR intervals), CVRR (Coefficient of Variation of the RR interval), SD1/SD2 (Standard Deviation 1/2 of short-term beat-to-beat from HRV).

Supplementary Table 4: Na^+-currents in acutely isolated cerebellar Purkinje cells.

	Voltage step	wt/wt	mut/wt	mut/mut	p-values (ANOVA on ranks/Dunn's test)
		n=27	n=33	n=12	
**CD**		208.1 ± 18.9	200.7 ± 22.6	121.3 ± 12.4*	0.011/<0.05
		n=25	n=30	n=12	
**$V_{1/2}$ activation [mV]**		-28.0 ± 1.3	-28.9 ± 0.9	-30.8 ± 1.7*	0.373/<0.05
		n=24	n=26	n=9	
**$V_{1/2}$ inactivation [mV]**		-57.9 ± 0.7	-58.7 ± 1.0	-54.0 ± 1.0	0.024
					**p-values** (two-way ANOVA/Tukey test)
		n≤14	n≤23	n≤12	
**A_{rel}**	-30 mV	0.48 ± 0.03	0.63 ± 0.03*	0.61 ± 0.03*	0.001/0.001/0.004
	-20 mV	0.33 ± 0.02	0.45 ± 0.01*	0.44 ± 0.03*	0.001/<0.001/0.008
	-10 mV	0.25 ± 0.02	0.33 ± 0.01	0.41 ± 0.04*	0.001/<0.001
	0 mV	0.21 ± 0.03	0.26 ± 0.02	0.28 ± 0.04	
**Tau_{fast}**	-30 mV	0.87 ± 0.08	0.98 ± 0.06	1.12 ± 0.07*	0.001/<0.001
	-20 mV	0.56 ± 0.02	0.67 ± 0.03	0.81 ± 0.07*	0.001/<0.001
	-10 mV	0.45 ± 0.02	0.54 ± 0.02	0.68 ± 0.02*	0.001/<0.001
	0 mV	0.38 ± 0.02	0.43 ± 0.02	0.56 ± 0.02*	0.001/0.02
**Tau_{slow}**	-30 mV	4.78 ± 0.56	6.52 ± 0.73	6.93 ± 1.09	
	-20 mV	2.98 ± 0.21	4.53 ± 0.51	3.94 ± 0.42	
	-10 mV	2.43 ± 0.33	3.67 ± 0.40	4.87 ± 0.79*	0.001/<0.012
	0 mV	2.06 ± 0.30	2.89 ± 0.52	0.68 ± 0.16	0.001
		n=20	n=25	n=9	
**$I_{15\ ms}/I_{Peak}$**		2.3 ± 0.4	3.1 ± 0.3	4.3 ± 0.8*	0.006/<0.05

Values are expressed as mean ± SEM.

Supplementary Table 5: Membrane properties of inhibitory and excitatory neurons recorded in brain slices of WT and *Scn1a*^{L1649Q} knock-in mice

	Cortex						Hippocampus					
	Inhibitory neurons			Excitatory neurons			Inhibitory neurons			Excitatory neurons		
	wt/wt	mut/wt	p-value	wt/wt	mut/wt	p-value	wt/wt	mut/wt	p-value	wt/wt	mut/wt	p-value
**V_m [mV]**	-70.1 ± 1.8	-66.6 ± 1.3	0.177	-70.0 ± 1.7	-70.1 ± 1.8	0.985	-68.8 ± 1.0	68.1 ± 2.7	0.599	-68.6 ± 1.1	-69.3 ± 1.6	0.405
**R_{in} [MΩ]**	171.7 ± 9.7	194.5 ± 12.3	0.167	149.4 ± 11.0	129.6 ± 8.8	0.173	173.6 ± 18.1	186.7 ± 30.3	0.694	148.5 ± 12.8	133.5 ± 10.0	0.364
**Rheobase [pA]**	52.9 ± 1.5	57.14 ± 4.6	0.099	69.4 ± 1.8	66.9 ± 1.7	0.556	63.6 ± 1.9	63.6 ±1.2	0.430	62.7 ± 1.8	63.7 ± 1.5	0.604

Values are expressed as mean ± SEM.

Supplementary Table 6: Nucleated patch recordings of hippocampal inhibitory neurons.

		wt/wt	mut/wt	p-value
	Voltage step	n=8	n=9	
**Persistent Na^+-current**	-30 mV	1.6 ± 0.4	2.4 ± 0.3	0.181
	-20 mV	1.5 ± 0.5	2.2 ± 0.5	0.340
	-10 mV	1.6 ± 0.48	2.9 ± 0.7	0.153
	0 mV	1.9 ± 0.6	2.9 ± 0.6	0.314
	10 mV	2.6 ± 0.6	3.0 ± 0.7	0.636
**Tau_h**	0 mV	0.31 ± 0.03	0.32 ± 0.01	0.599
**Tau_{Rec}**	-100 mV	4.8 ± 1.0	7.4 ± 2.4	0.247

Values are expressed as mean ± SEM.

Supplementary Table 7: Physiological monitoring during in vivo CSD recordings.

	**All animals**			**2 months old animals**			**9 months old animals**		
	**wt/wt**	**mut/wt**	**p-value**	**wt/wt**	**mut/wt**	**p-value**	**wt/wt**	**mut/wt**	**p-value**
**bodyweight, g**	28 [25, 37]	28.5 [25, 36.75]	0.81	25 [22.5, 26.5]	25 [22, 25]	0.78	37 [30, 39]	36 [32, 40]	0.70
**endtidal pCO_2, mmHg**	20.5 [18.39, 23.13]	20.4 [18.33, 23.07]	0.82	20.4 [17.51, 21.08]	19.7 [18.17, 22.37]	0.91	20.5 [19.07, 25.14]	22.3 [18.59, 20.88]	0.66
**MAP, mmHg**	66.6 [62.56, 71.34]	68.9 [60.78, 77.25]	0.65	66.3 [62.75, 68.25]	65.9 [56.81, 68.88]	0.53	67.8 [63.72, 75.65]	77.2 [69.57, 89.66]	0.20
**SpO_2, %**	93.6 [90, 97]	95.0 [88.1, 96]	0.72	93.7 [91, 97]	95.0 [87.52, 95.85]	0.69	92.0 [88, 97.5]	95.0 [88.42, 96]	0.88
**temperature, °C**	37.4 [37.35, 37.47]	37.4 [37.3, 37.45]	0.47	37.4 [37.05, 37.46]	37.4 [37.18, 37.46]	1	37.4 [37.41, 37.49]	37.4 [37.31, 37.44]	0.26

Values are expressed as median [1st quartile, 3rd quartile]. No significant differences (Wilcoxon rank sum test). MAP (mean arterial pressure), SpO2 (peripheral oxygen saturation)

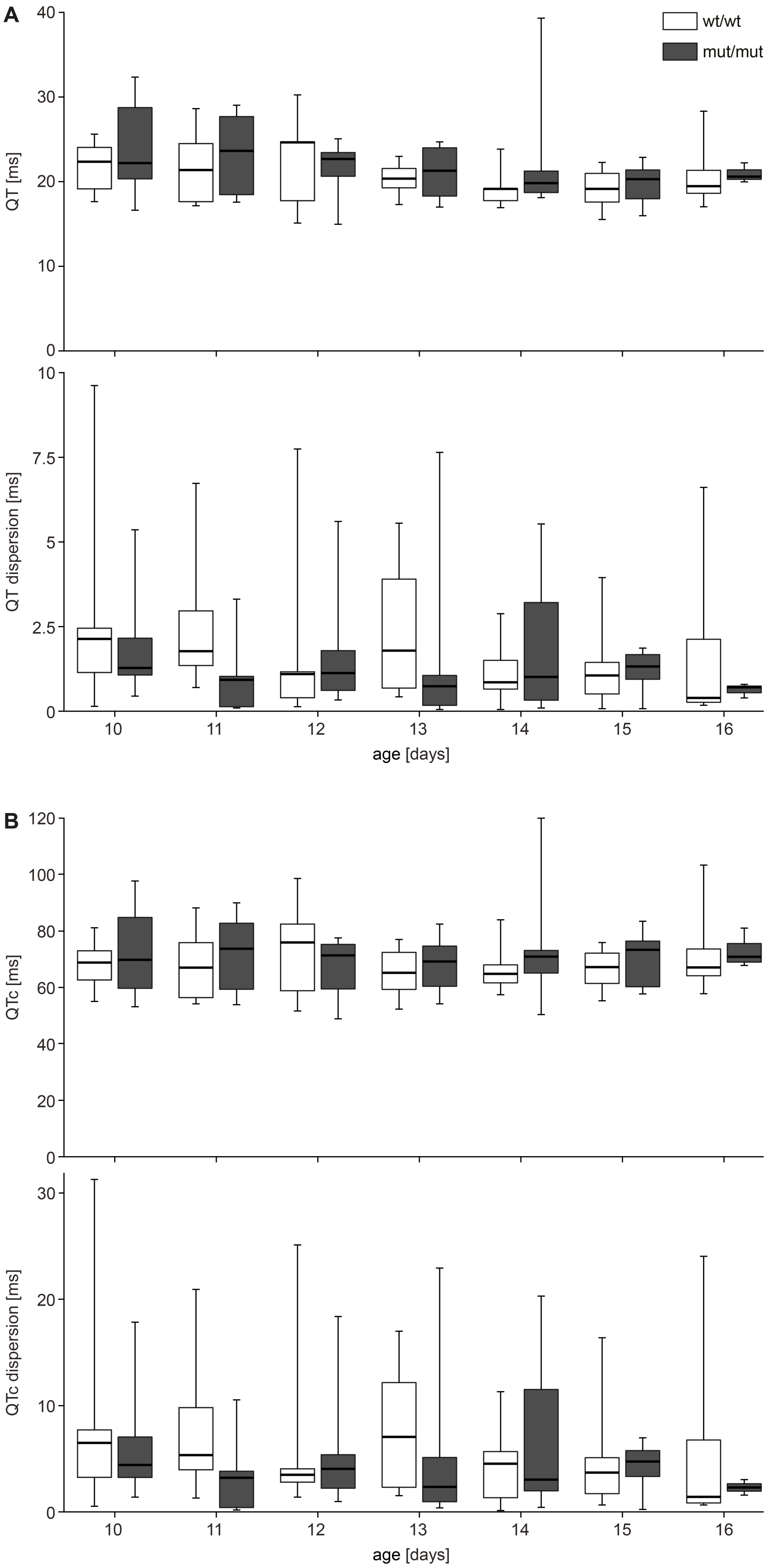

Supplementary Figure 1: Electrocardiography of homozygous *Scn1a*^{L1649Q} knock-in mice
QT (panel A) and QTc analysis (panel B) of homozygous (grey boxes) and wildtype (white boxes) animals from P10 to P16. No significant differences (group sizes: wt/wt, n=9, mut/mut, n=10; Wilcoxon rank sum test).

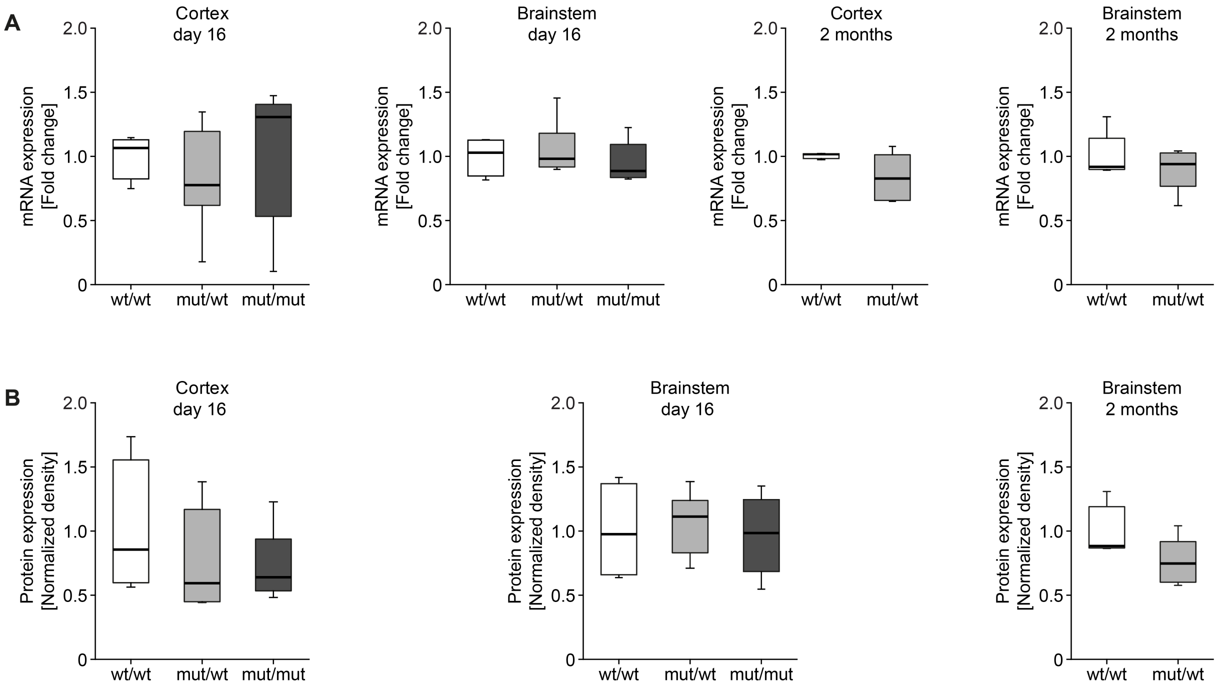

Supplementary Figure 2: *Scn1a* mRNA and protein levels in *Scn1a*^{L1649Q} knock-in animals. (A) mRNA expression of *Scn1a* in cortex and brainstem on postnatal day 16 and 2 months of age. Values express relative fold change normalized to wildtype. There was no difference among the groups (Group sizes: p16: wt/wt, n=6; mut/wt, n=4; mut/mut, n=5; 2 months: wt/wt, n=5; mut/wt, n=5; Mann-Whitney Rank Sum Test). **(B)** Western blot analysis of membrane protein fractions prepared from cortex and brainstem of *Scn1a*^{L1649Q} knock-in mice at the age of 16 days (wildtype, heterozygous and homozygous littermates) and from brainstem at the age of 2 months (wildtype and heterozygous littermates) using $Na_V1.1$ - and beta-actin-antibodies. Quantification of $Na_V1.1$ level is relative to actin levels. Equal $Na_V1.1$ protein level in P16 animals and a trend to lower levels in brainstem samples of heterozygous *Scn1a*^{L1649Q} knock-in mice at the age of 2 months ($p<0.1$; Group sizes: p16: wt/wt, n=6; mut/wt, n=4; mut/mut, n=5; 2 months: wt/wt, n=5; mut/wt, n=5; Mann-Whitney Rank Sum Test).

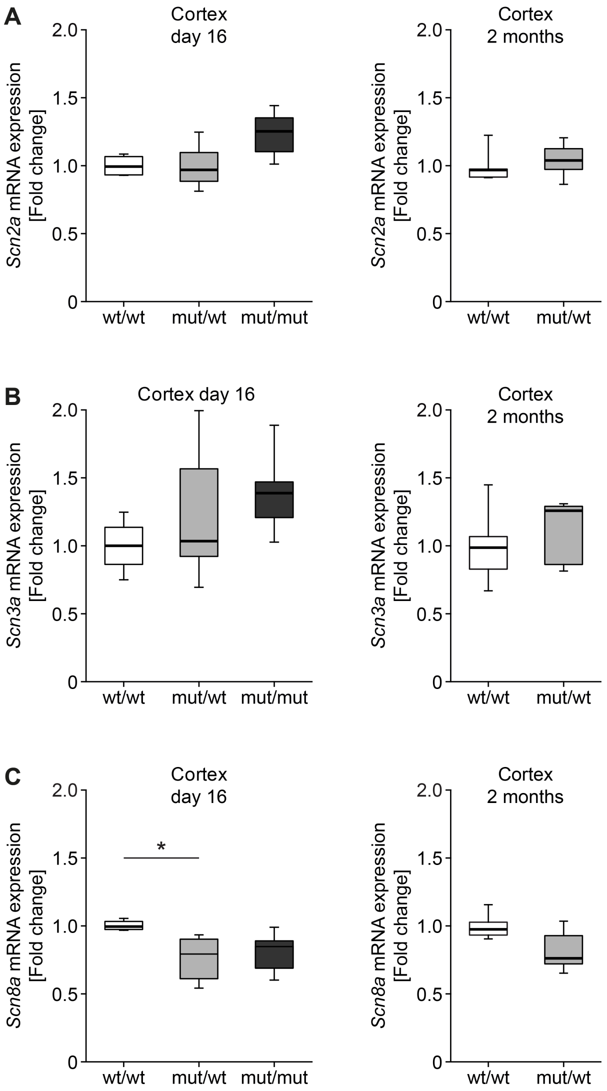

Supplementary Figure 3: mRNA levels of voltage gated sodium channels in *Scn1a*^{L1649Q} knock-in animals. mRNA expression of voltage-gated sodium channels in cortex on postnatal day 16 and 2 months of age. Values express relative fold change normalized to GAPDH and normalized to wildtype. Group sizes: p16: wt/wt, n=4; mut/wt, n=6; mut/mut, n=5; 2 months: wt/wt, n=5; mut/wt, n=5; Mann-Whitney Rank Sum Test, p-values given for: p16 wt/wt vs. mut/wt and wt/wt vs. mut/mut and 2 months wt/wt vs mut/wt **(A)** mRNA expression of *Scn2a.* There was no difference among the groups (p=0.93, p=0.063 and p=0.69). **(B)** mRNA expression of *Scn3a.* There was no difference among the groups (p=0.53, p=0.16 and p=0.69). **(C)** mRNA expression of *Scn8a*. mRNA level was reduced in p16 heterozygous animals. (p=0.0061, p=0.0635 and p=0.15).

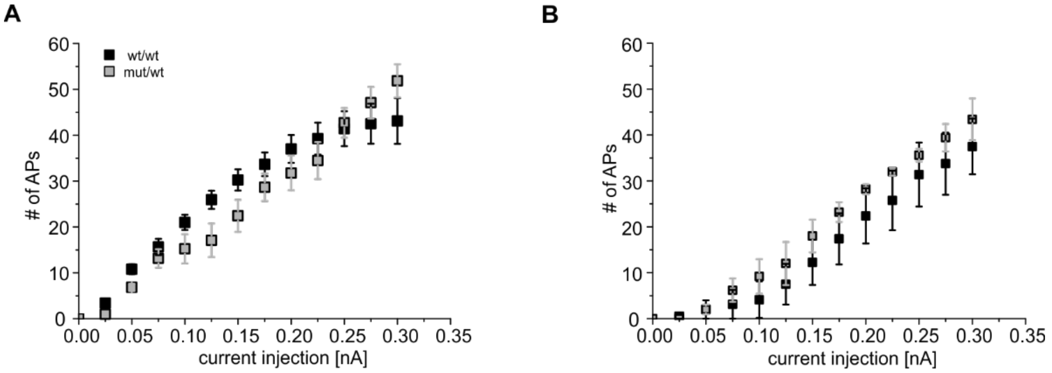

Supplementary Figure 4: Firing rate of regular spiking inhibitory neurons in the hippocampal *stratum oriens* of the CA1 region was not altered in slices of heterozygous animals compared to wildtype littermates. (A) Number of APs per trace plotted vs size of current injection for regular spiking inhibitory neurons of hippocampal CA1 region. The area under the curve was not significantly different between mut/wt and wt/wt animals. (wt/wt: n=16 cells from 6 animals (16/6); mut/wt: 15/5; Mann-Whitney Rank Sum Test). **(B)** Number of APs per trace plotted vs size of current injection for regular spiking inhibitory neurons of cortical layer 4. The area under the curve was not significantly different between mut/wt and wt/wt animals. (wt/wt: n=16 cells from 6 animals (16/6); mut/wt: 15/5; Mann-Whitney Rank Sum Test).

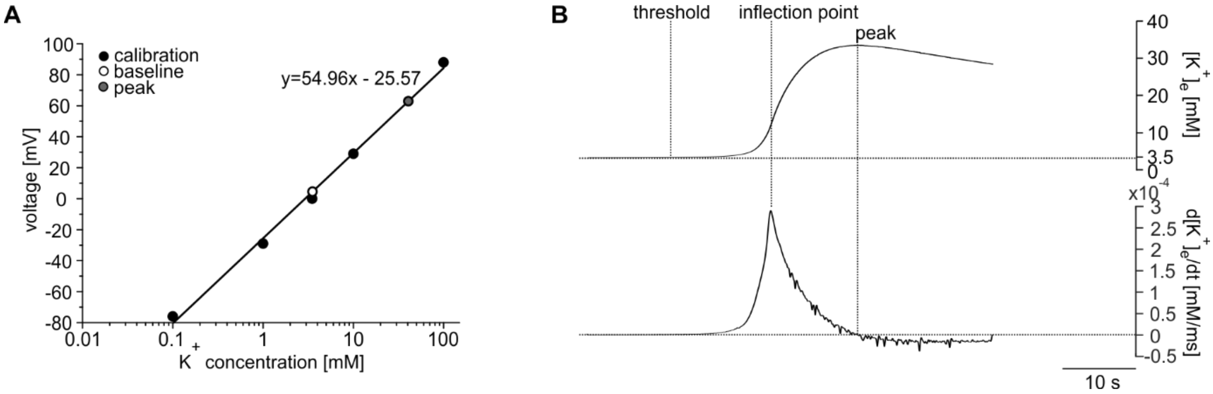

Supplementary Figure 5: Representative calibration curve for K^+ selective electrodes and identification of the inflection-point and CSD threshold using the first derivative of the $[K^+]_e$ recording. (A) Slope of the calibration curve was 54.96. In addition, the value for the baseline potential (white dot) and the peak potential (grey dot) are shown. **(B)** Averaged $[K^+]_e$ dynamics before and during CSD in slices of mut/wt animals (top) as well as 1st derivative of the curve (bottom). Dotted lines indicate the points at which (i) the K^+ signal left the baseline (threshold of 0.1 mM), (ii) the peak of the 1st derivative showing the inflection-point of the $[K^+]_e$ curve and (iii) the peak of the $[K^+]_e$ trace.

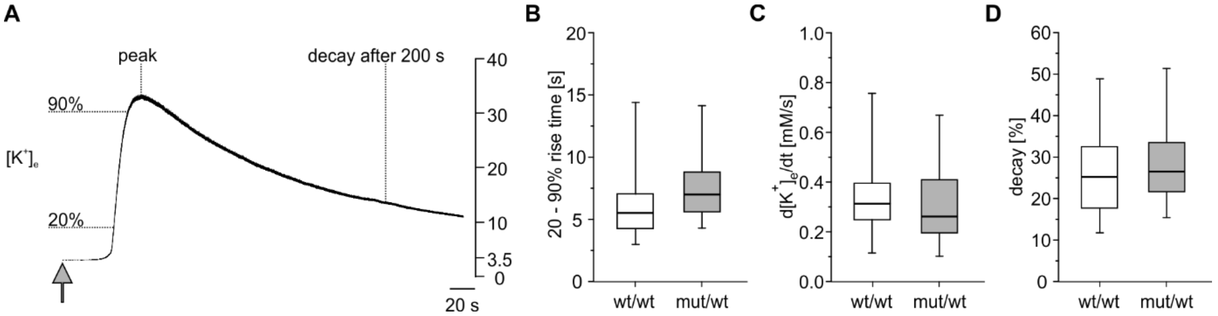

Supplementary Figure 6: Rise time and decay of the $[K^+]_e$ dynamics. (A) Representative $[K^+]_e$ signal showing the time of KCl puff (arrow), the 20-90% rise time, the peak of the $[K^+]_e$ signal and the decay 200s after the peak. **(B)** The 20-90% rise time was not different between slices of wt/wt and mut/wt animals (wt/wt: (22/10); mut/wt: (31/11); Mann-Whitney Rank Sum Test). **(C)** The maximum rising speed of $[K^+]_e$, determined as the peak of the 1st derivative, did not differ between wt/wt and mut/wt (wt/wt: (22/10); mut/wt: (31/11); Mann-Whitney Rank Sum Test). **(D)** The decay of $[K^+]_e$ signal 200s after the peak of the signal, determined as the relative $[K^+]_e$ compared to the peak $[K^+]_e$, was not different between wt/wt and mut/wt animals (wt/wt: (22/10); mut/wt: (31/11); Mann-Whitney Rank Sum Test).

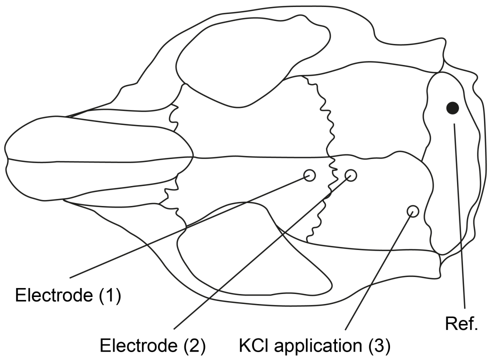

Supplementary Figure 7: Schematic representation of electrode placement for in vivo CSD recordings

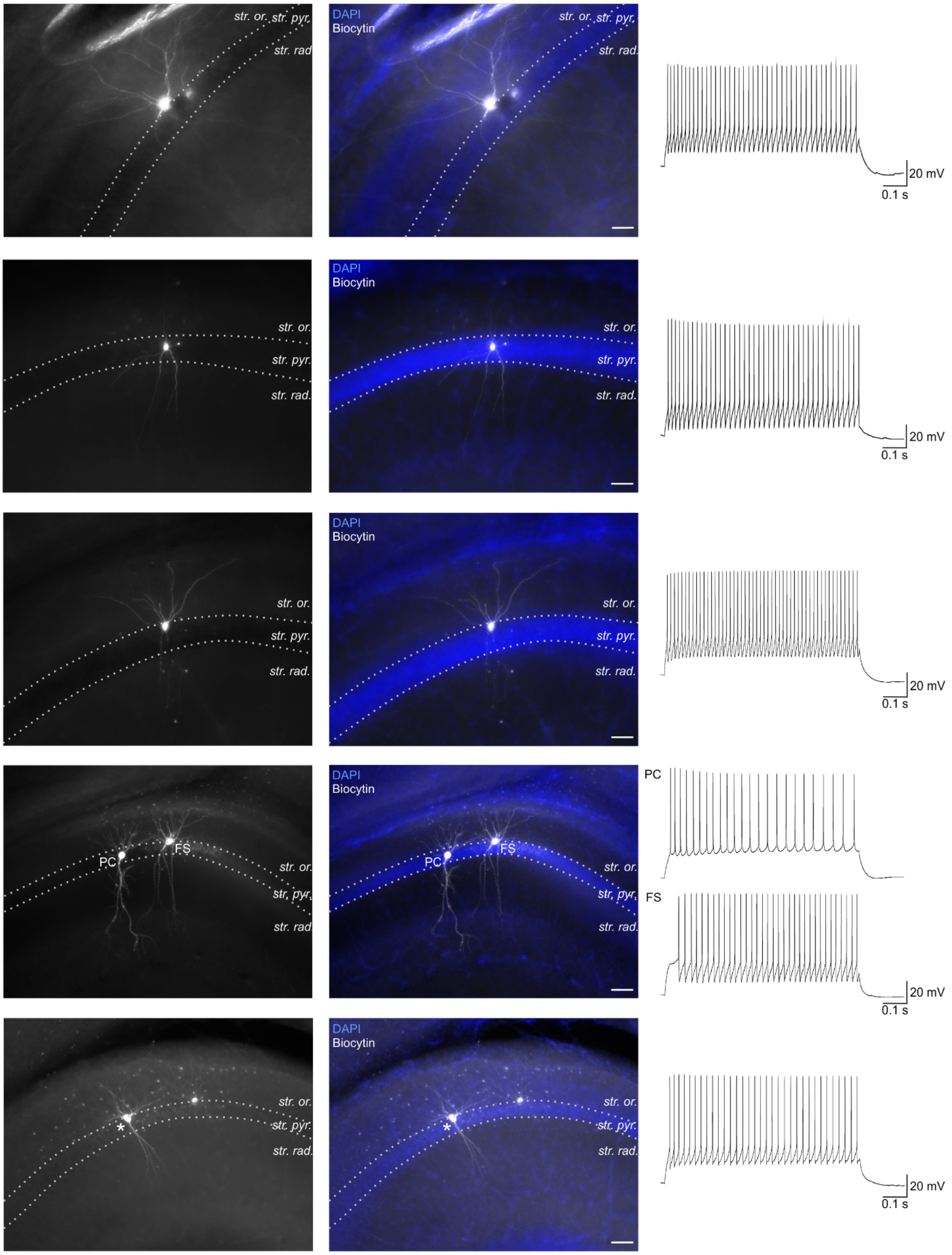

Supplementary Figure 8: Representative hippocampal neurons filled with biocytin. Pictures of five recorded fast spiking inhibitory neurons (FS) and one pyramidal neuron (PC) in the hippocampal CA1 region. Cells were filled with biocytin (white) and stained against DAPI (blue). Scale bars, 100 µm, *str. or.*: *stratum oriens*, *str. pyr.*: *stratum pyramidale*, *str. rad.*: *stratum pyramidale*. Firing behaviour of recorded and biocytin filled neurons is shown on the right.
